# Pleiotropic Effects of the P5-Type ATPase SpfA on Stress Response Networks Contribute to Virulence in the Pathogenic Mold Aspergillus fumigatus

**DOI:** 10.1128/mBio.02735-21

**Published:** 2021-10-19

**Authors:** José P. Guirao-Abad, Martin Weichert, Ginés Luengo-Gil, Sarah Sze Wah Wong, Vishukumar Aimanianda, Christina Grisham, Nikita Malev, Shivani Reddy, Laura Woollett, David S. Askew

**Affiliations:** a Department of Pathology and Laboratory Medicine, University of Cincinnati College of Medicine, Cincinnati, Ohio, USA; b Clinical Analysis and Pathology Department, Group of Molecular Pathology and Pharmacogenetics, Instituto Murciano de Investigación Biosanitaria (IMIB), Hospital Universitario Santa Lucía, Cartagena, Spain; c Institut Pasteur, Molecular Mycology Unit, CNRS, UMR2000, Paris, France; University of Texas Health Science Center

**Keywords:** *Aspergillus fumigatus*, UPR, HacA, ER stress, Spf1, SpfA, SERCA, P5-type ATPases, cell wall, lipid metabolism, sterols, redox homeostasis, lipid homeostasis, redox balance

## Abstract

Aspergillus fumigatus is a human-pathogenic mold that extracts nutrients from the environment or from host tissues by secreting hydrolytic enzymes. The ability of A. fumigatus to adjust secretion levels in proportion to demand relies on the assistance of the unfolded protein response (UPR), an adaptive stress response pathway that regulates the unique protein-folding environment of the endoplasmic reticulum (ER). The P5-type ATPase Spf1 has recently been implicated in a novel mechanism of ER homeostasis that involves correcting errors in ER-membrane protein targeting. However, the contribution of this protein to the biology of A. fumigatus is unknown. Here, we employed a gene knockout and RNA sequencing strategy to determine the functional role of the A. fumigatus gene coding for the orthologous P5 ATPase SpfA. The data reveal that the *spfA* gene is induced by ER stress in a UPR-dependent manner. In the absence of *spfA*, the A. fumigatus transcriptome shifts toward a profile of altered redox and lipid balance, in addition to a signature of ER stress that includes *srcA*, encoding a second P-type ATPase in the ER. A Δ*spfA* deletion mutant showed increased sensitivity to ER stress, oxidative stress, and antifungal drugs that target the cell wall or plasma membrane. The combined loss of *spfA* and *srcA* exacerbated these phenotypes and attenuated virulence in two animal infection models. These findings demonstrate that the ER-resident ATPases SpfA and SrcA act jointly to support diverse adaptive functions of the ER that are necessary for fitness in the host environment.

## INTRODUCTION

The filamentous fungus Aspergillus fumigatus is an opportunistic pathogen of humans and animals that propagates itself by releasing conidia (spores) into the atmosphere. The fungus is found worldwide, so the inhalation of these conidia is a daily occurrence for most individuals (1). Although inhaled conidia are effectively cleared in the healthy human population, patients who are immunosuppressed or who have preexisting lung structural disease possess weakened pulmonary clearance defenses, which allows the conidia to germinate into the invasive hyphal form of the organism. After traversing the epithelial barrier, the hyphae damage the lung parenchyma, resulting in life-threatening pneumonia that may spread systemically. Despite advances in antifungal therapy, invasive aspergillosis continues to be associated with high rates of morbidity and mortality and has become one of the most feared opportunistic infections in transplant units worldwide (2–5). Current evidence suggests that the virulence of A. fumigatus is due, in part, to a high degree of adaptability, endowing the fungus with the ability to withstand harsh environmental conditions, including exposure to antifungal drugs (1). This underscores the need for a more detailed understanding of the genes and pathways involved in stress resistance so that novel targets of vulnerability can be identified.

Filamentous fungi rely on an extensive endoplasmic reticulum (ER) network to support the delivery of new plasma membrane and cell wall material to hyphal tips as well as to secrete hydrolytic enzymes into the extracellular milieu (6). Since high concentrations of proteins in the ER lumen increase the risk for illegitimate interactions that may arise during protein folding, eukaryotic cells employ a series of adaptive signaling mechanisms to ensure that proteins achieve their native conformation. The unfolded protein response (UPR) is one such signaling network that plays a major role in the adaptation of eukaryotic organisms to stress caused by an increase in the level of unfolded or misfolded proteins in the ER. In mammals, there are three mechanistically distinct branches of the UPR, each controlled by a separate ER transmembrane sensor: IRE1, PERK, and ATF6 (7). IRE1 directs the most ancient branch of the UPR and represents the sole ER sensor in the fungal kingdom. All known species homologs of IRE1 possess an ER luminal unfolded protein-sensing domain and a cytoplasmic portion containing two enzymatic domains: a kinase and an endoribonuclease (RNase). The ortholog of IRE1 in A. fumigatus, known as IreA, follows the paradigm of fungal UPR signaling established in Saccharomyces cerevisiae (8, 9). The IreA protein triggers the pathway by regulating the splicing of a precursor mRNA known as *hacA*^u^ (u for uninduced), which is transcribed from the *hacA* gene. In response to the accumulation of abnormally folded proteins in the ER lumen, the IreA RNase removes an unconventional intron from the *hacA*^u^ mRNA, thereby converting it into the induced form of the mRNA, *hacA*^i^. This splicing reaction generates a shift in the open reading frame, which specifies the translation of a bZIP transcription factor called HacA. After translocating to the nucleus, HacA orchestrates the upregulation of UPR target genes that are necessary to augment ER protein-folding capacity. Since many gene products that traffic through the secretory pathway also support phenotypic traits that are tightly linked to pathogenicity, the UPR has become increasingly recognized as a virulence signaling hub for diverse species of fungi that cause disease in both humans and plants (7–17).

The UPR is best known for its ability to upregulate the expression of chaperones that assist protein folding in the ER lumen. To ensure that sufficient quantities of Ca^2+^ are available to support Ca^2+^-dependent chaperones, the A. fumigatus UPR coordinately upregulates the expression of ER and Golgi P-type Ca^2+^ ATPases, which are necessary to support virulence (18). P-type ATPases are a large class of transporters that cycle between phosphorylated and dephosphorylated states, which provides the energy that is necessary for the translocation of diverse substrates, including various metal ions (Na^+^, K^+^, Ca^2+^, and Mn^2+^) and lipids (19–21). Members of this family arose by a complex pattern of gene duplication events (22) and are phylogenetically conserved (23), grouped into five subfamilies based on their sequence homology and preferred transport substrates. The P1 to P4 ATPases are the best characterized, with established functions as cation or lipid transporters (19). However, the substrate specificity and biological function of the orphan P5 ATPases have been more elusive (24). In S. cerevisiae, the single P5 ATPase, Spf1 (sensitivity to *Pichia farinosa* killer toxin 1), localizes to the ER membrane, and its deletion results in pleiotropic effects on lipid homeostasis, Ca^2+^ and Mn^+2^ transport, and the localization of some tail-anchored proteins (25–30). Spf1 has recently been implicated in a novel ER quality control mechanism that maintains ER homeostasis by correcting mistakes in protein targeting to the ER membrane (31). The size and topology of the substrate-binding pocket of Spf1 are unusually large relative to the structure of other P-type ATPases that transport ions and lipids (31). This suggests that Spf1 has a fundamentally different substrate specificity, which allows it to dislocate misinserted proteins from the ER membrane. Among the pathogenic fungi, the diverse functions provided by Spf1 are important for virulence in the human commensal and opportunistic pathogen Candida albicans (32), the entomopathogenic fungus Beauveria bassiana (33), and the hemibiotrophic plant pathogen Pyricularia oryzae (previously known as Magnaporthe oryzae) (34). However, the contribution of this P5 ATPase to the biology and virulence of A. fumigatus is unknown.

Here, we demonstrate that the A. fumigatus *spfA* gene, which encodes the ortholog of Spf1, is induced by ER stress in a UPR-dependent manner and that the encoded protein localizes to the ER. A transcriptomic analysis revealed over a thousand genes with altered expression patterns in response to *spfA* deletion, which correlated with increased susceptibility of the mutant to agents that disrupt ER homeostasis, cell wall and membrane homeostasis, and redox balance. Previously identified components of a core UPR transcriptome in A. fumigatus were upregulated in the Δ*spfA* mutant (8), including the *srcA* gene, which encodes a second P-type ATPase that also localizes to the ER membrane. The combined loss of the *spfA* and *srcA* genes exacerbated many of the phenotypes displayed by the Δ*spfA* mutant and attenuated the virulence of the fungus. These findings indicate that the SpfA and SrcA ATPases act in concert to support diverse adaptive functions of the ER.

## RESULTS

### The A. fumigatus gene *spfA* is induced by ER stress in a UPR-dependent manner.

**FIG 1:**
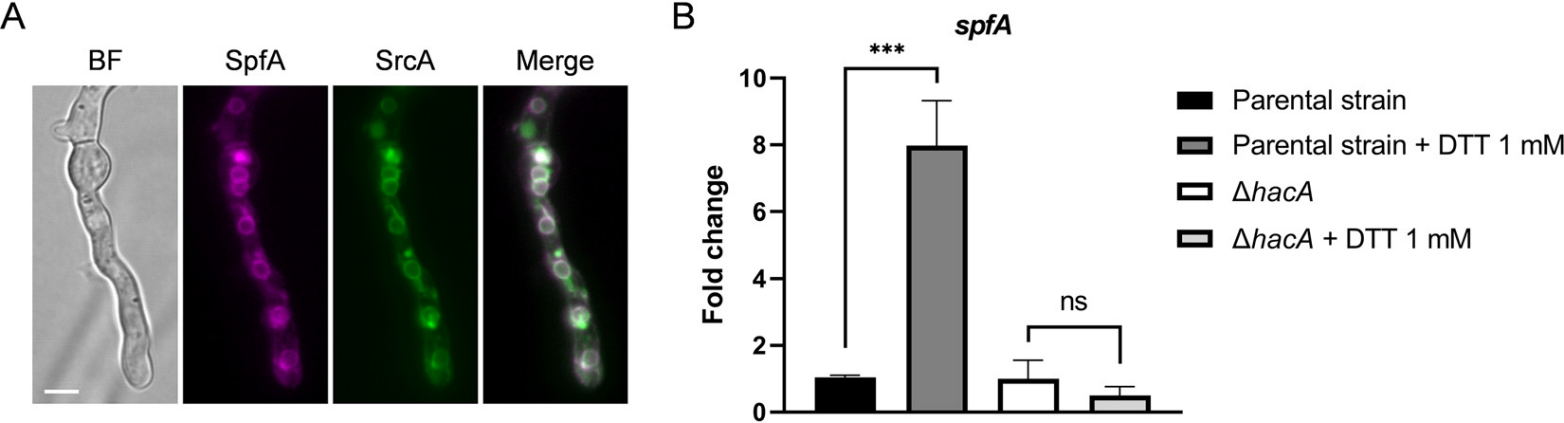
The *spfA* gene is a downstream target of the UPR in A. fumigatus and encodes a protein that localizes to the ER membrane. (A) *In situ* tagging of the *spfA* gene with *mrfp1* reveals colocalization of the SpfA-mRFP1 fusion protein (false colored in magenta) with the fluorescent signals of the ER membrane-resident Ca^2+^ pump SrcA labeled with eGFP (green). The bright-field (BF) and fluorescence microscopy images show a germling grown for 17 h at 30°C in liquid AMM. Bar, 5 μm. (B) RT-qPCR analysis of *spfA* gene expression in cultures of the Δ*hacA* mutant and its parental strain KU70 grown in liquid YG medium for 16 h at 37°C at 200 rpm. ER stress was induced by treatment with 1 mM DTT for 1 h prior to the extraction of mRNA. Values represent the means ± standard deviations (SD) from three technical replicates (****, *P < *0.0001; ns, not significant [by one-way ANOVA with Tukey’s *post hoc* test]).

A search of the A. fumigatus genome database using the Saccharomyces cerevisiae P5 ATPase Spf1 protein as the query identified the uncharacterized protein Afu3g13790 (SpfA) as the closest homolog (52% identity and 68% similarity). A comparison of A. fumigatus SpfA with known protein signatures using InterPro 84.0 (35) revealed SpfA to be a member of P-type ATPase subfamily V (IPR006544). P5 ATPases are further divided into two subfamilies: P5A-type ATPases localize to the ER membrane, whereas P5B-type ATPases are vacuolar or lysosomal membrane-associated proteins (24, 36). An alignment of SpfA with other species homologs of Spf1 revealed conservation of the 12 predicted transmembrane domains, including the extended N-terminal domain (NTD) preceding the A domain that is shared with the P5A ATPase class (see Fig. S1 in the supplemental material). Since orthologs of Spf1 in other fungal species have been shown to have an ER localization (27, 34), we tagged the *spfA* gene *in situ* with mRFP1 (monomeric red fluorescent protein 1) and colocalized the protein with the enhanced green fluorescent protein (eGFP)-tagged SERCA-type Ca^2+^ ATPase SrcA (SERCA: sarco/endoplasmic reticulum calcium-ATPase) (18). Consistent with reports of orthologs in other filamentous fungi (37, 38), SrcA-enhanced GFP (SrcA-eGFP) colocalized with an ER-specific dye (Fig. S2A). As expected, the subcellular localization of the SpfA-mRFP1 fusion protein largely overlapped that of SrcA-eGFP (Fig. 1A). Taken together, these findings indicate that SpfA is an ER-resident protein in A. fumigatus and represents the ortholog of the Spf1 P5A-type ATPase.

10.1128/mBio.02735-21.1 FIG S1 Multiple-sequence alignment of SpfA/Spf1 orthologs. The sequence of A. fumigatus SpfA is aligned with the sequences of Spf1 proteins from different species. The alignment was generated with the Web tools T-coffee (http://tcoffee.crg.cat/apps/tcoffee/do:regular) and Boxshade (https://embnet.vital-it.ch/software/BOX_form.html). Black shading indicates conserved residues, and gray shading indicates conservative changes. The domains and motifs were annotated as described previously by McKenna et al. (31). TM, transmembrane; NTD, N-terminal domain; A-domain, cytosolic actuator domain; N-domain, nucleotide-binding domain; P-domain, phosphorylation domain; yellow box, conserved DKTG phosphorylation site; blue box, conserved (S/T)GES motif; red box, conserved PP(E/D)LP motif. Download FIG S1, PDF file, 0.5 MB. Copyright © 2021 Guirao-Abad et al. 2021 Guirao-Abad et al. https://creativecommons.org/licenses/by/4.0/ This content is distributed under the terms of the Creative Commons Attribution 4.0 International license.

10.1128/mBio.02735-21.2 FIG S2 Analysis of strains expressing SrcA-eGFP and SpfA-mRFP. (A) *In situ* tagging of the *srcA* gene with *egfp* reveals colocalization of the SrcA-eGFP fusion protein (green) with the blue fluorescent signal of the ER-tracker blue-white DPX dye (Invitrogen). The bright-field (BF) and fluorescence microscopy images show a germling grown for 15 h at 37°C in liquid AMM. Bar, 5 μm. (B) Strains expressing fluorescently tagged proteins show normal stress responses. Serial 10-fold dilutions of conidia from the indicated strains were spotted onto AMM plates containing EGTA, tunicamycin (TM), hygromycin B (HygB), calcofluor white (CW), and caspofungin (CSF). Plates were incubated for 2 days at 37°C. (C) Strains expressing fluorescently tagged proteins grow normally. The colony morphology of the indicated strains on YG or YPD medium after 3 days of growth at 37°C was assessed. Download FIG S2, JPG file, 1.6 MB. Copyright © 2021 Guirao-Abad et al. 2021 Guirao-Abad et al. https://creativecommons.org/licenses/by/4.0/ This content is distributed under the terms of the Creative Commons Attribution 4.0 International license.

We have previously shown that two P2-type ATPases that transport Ca^2+^ into the ER and Golgi apparatus are integral components of the adaptive response to a disturbance of ER homeostasis (18). To determine how the A. fumigatus *spfA* gene responds to ER stress, cultures were treated for 1 h with dithiothreitol (DTT), a strong reducing agent that triggers acute protein-unfolding stress by breaking disulfide bonds. Analysis of gene expression by reverse transcription-quantitative PCR (RT-qPCR) revealed the induction of the *spfA* mRNA following DTT treatment. However, DTT did not induce *spfA* expression in a Δ*hacA* mutant that lacks the transcription factor that is essential for UPR target gene activation (Fig. 1B), suggesting that the *spfA* gene is a transcriptional target of the canonical UPR pathway in A. fumigatus.

### Transcriptomic analysis reveals that the loss of *spfA* induces acute ER stress in A. fumigatus.

**FIG 2:**
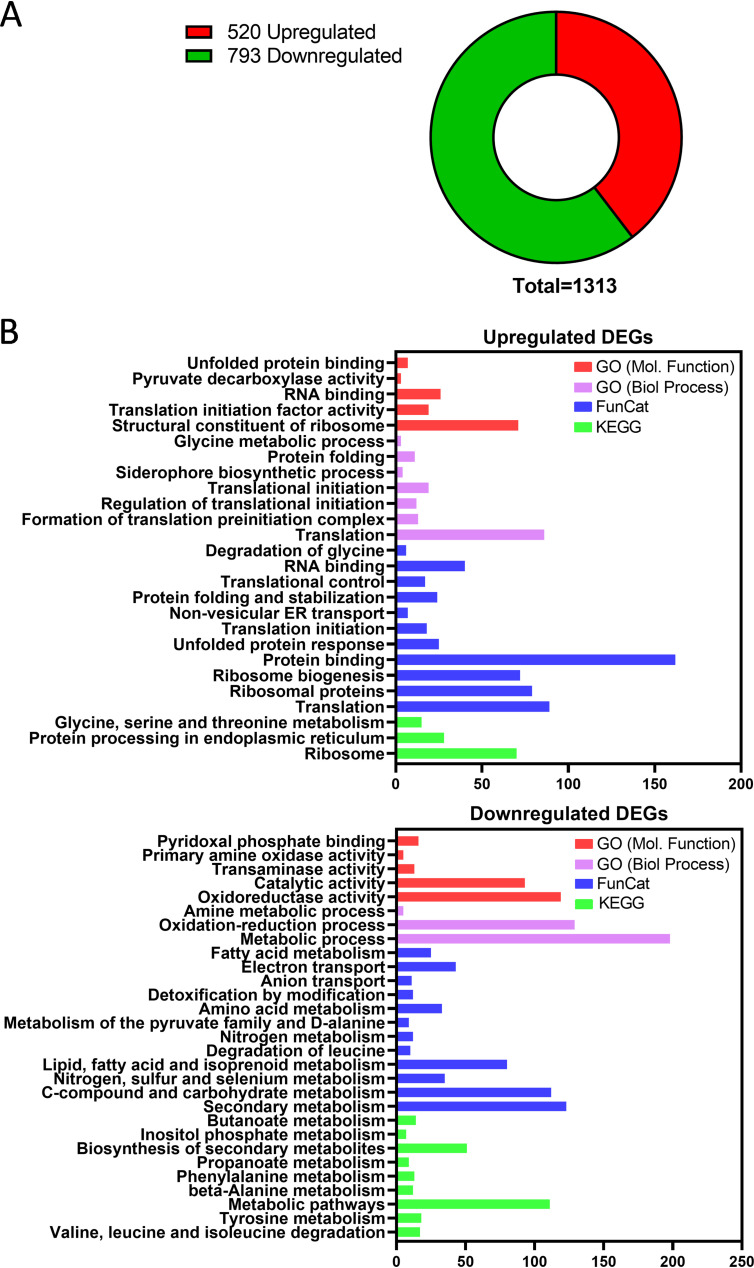
Analysis of differentially expressed genes (DEGs) in the absence of *spfA*. (A) Pie chart summarizing the total number of DEGs (Bonferroni *P* value of <0.05) in the Δ*spfA* mutant. (B) Enrichment analysis of upregulated and downregulated DEGs using Gene Ontology, FunCat, and KEGG. Overrepresented categories adjusted with the Benjamini-Yuketieli procedure are shown.

The *spf1* gene has been studied in a variety of species, but the impact of its deletion on the fungal transcriptome is unknown. To address this gap in knowledge, a Δ*spfA* mutant of A. fumigatus was constructed by homologous recombination with a gene knockout cassette and subsequently reconstituted by site-specific reintegration of the *spfA* gene (Fig. S3). The Δ*spfA* mutant, which conidiated normally, showed a mild radial growth defect at 37°C, and no further growth impairment was observed at 45°C (Fig. S4A). Total RNA was isolated from cultures grown in rich medium (yeast extract-glucose [YG]) at 37°C, and RNA sequencing (RNA-Seq) was used to compare the transcriptional profile of the Δ*spfA* mutant to that of its parental strain KU80. The experiment was performed in triplicate, and mRNAs were considered to be differentially expressed in the Δ*spfA* mutant if their abundance was statistically different from those in the KU80 reference strain following the application of a stringent Bonferroni *post hoc* test. A total of 1,313 differentially expressed genes (DEGs) were identified by this approach, 520 of which were upregulated and 793 of which were downregulated in the Δ*spfA* mutant relative to KU80 (Fig. 2A). Computational analysis of the DEGs was performed using the FungiFun2 Web tool (39) that integrates the following functional ontologies: Gene Ontology (GO) (40), Functional Catalogue (FunCat) (41), and the Kyoto Encyclopedia of Genes and Genomes (KEGG) (42). A summary of the categories that were found to be overrepresented using each of the above-mentioned annotation tools is presented in Fig. 2B. All four analytical methods identified two major groups of upregulated genes. The first centered around genes with functions that are known to play a role in the UPR and ER quality control or to impact ER function in some other manner. These included categories of unfolded protein binding, protein folding, protein stabilization, the unfolded protein response, nonvesicular ER transport, and protein processing in the ER. The second major group of upregulated genes encodes proteins that are broadly involved with the translational machinery, involving categories of translation initiation and control as well as ribosome biogenesis and structure. This suggests that the absence of SpfA creates an environment that drives the need for increased protein biosynthetic capacity, allowing the fungus to achieve a new homeostatic state that is compatible with the adverse pleiotropic effects caused by *spfA* deletion.

10.1128/mBio.02735-21.3 FIG S3 Validation of the Δ*spfA* and Δ*spfA*/Δ*srcA* mutants of A. fumigatus. (A, top) PCR confirmation of the Δ*spfA* strain and the complemented mutant. The *spfA* gene knockout was confirmed using primer pairs that generate PCR products specific for the wild-type locus (1390/1391, 1392/1468, and 1022/1027) and/or the deletion (1022/1027). Complementation of the Δ*spfA* mutant was confirmed with primer pairs that detect the site-specific reintegration of the *spfA* gene (1183/1468 and 1287/1139). (Bottom) Schematic representation of the primer locations. The left-arm (LA) and right-arm (RA) regions of the *spfA* wild-type locus (orange) undergo homologous recombination with the knockout construct p706, leaving behind a *six* site after β-recombinase-directed excision of the marker module from the locus, as detailed in Materials and Methods. Reintegration of the *spfA* gene into the mutant was accomplished by recombination of the vector p713 with an intergenic region (IR). (B, top) PCR confirmation of the Δ*spfA*/Δ*srcA* mutant and the complemented double mutant. In addition to primer combinations specific for the *spfA* wild-type and/or deleted locus used in panel A, a primer pair specific for the status of the *srcA* gene (1073/1012) was used to confirm the absence of both the *spfA* and *srcA* genes in the double mutant. Complementation of the Δ*spfA*/Δ*srcA* mutant with a copy of the *spfA* gene targeted to the IR was confirmed by PCR as shown in panel A. (Bottom) Schematic of the primer locations for the detection of the *srcA* wild-type or deletion locus. The left-arm and right-arm regions (green) of the *srcA* locus were originally used for the deletion of this gene (18). Download FIG S3, JPG file, 2.7 MB. Copyright © 2021 Guirao-Abad et al. 2021 Guirao-Abad et al. https://creativecommons.org/licenses/by/4.0/ This content is distributed under the terms of the Creative Commons Attribution 4.0 International license.

10.1128/mBio.02735-21.4 FIG S4 Loss of *spfA* is associated with a minor growth defect. (A) Conidia from the indicated strains were plated onto YG medium, and radial growth was monitored after 3 days of growth at 37°C and 45°C. (B) Colony morphology of the indicated strains on IMA, YG medium, or AMM after 3 days of growth at 37°C. Download FIG S4, JPG file, 1.7 MB. Copyright © 2021 Guirao-Abad et al. 2021 Guirao-Abad et al. https://creativecommons.org/licenses/by/4.0/ This content is distributed under the terms of the Creative Commons Attribution 4.0 International license.

**FIG 3:**
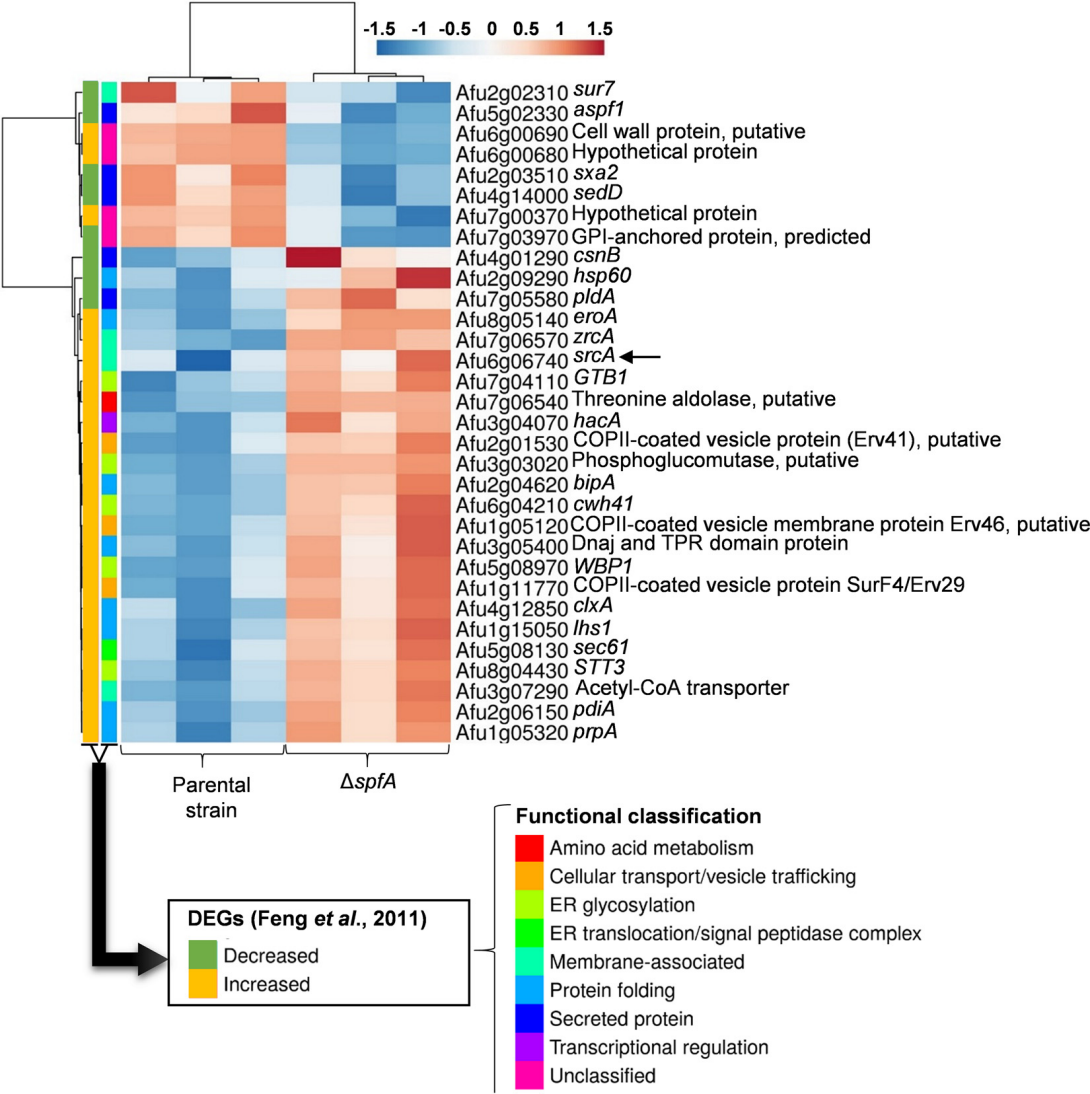
Heat map comparison of published UPR target genes with the DEGs identified in the Δ*spfA* mutant. The two vertical columns on the left indicate the functional classification of a core data set of acute ER stress response genes reported previously by Feng et al. (8). Horizontal bars on the right represent those DEGs identified in the Δ*spfA* mutant that overlap this core data set of UPR-responsive genes. The arrow highlights the A. fumigatus gene *srcA* (Afu6g06740). Data from three biological replicates per strain are shown. The log_2_ values of the fold changes in expression (log_2_FC) are represented as variations in the intensity of the color from blue (downregulated) to red (upregulated). Genes with similar expression values are grouped by hierarchal clustering using ClustVis (https://biit.cs.ut.ee/clustvis/). GPI, glycosylphosphatidylinositol.

We previously analyzed the transcriptional response of A. fumigatus to two agents that produce ER stress by different mechanisms: DTT by reducing disulfide bonds and tunicamycin (TM) by impairing N-linked glycosylation (8). Since each agent has additional effects unrelated to ER stress, those DEGs that were shared between the two treatments were defined as responsive to acute ER stress and thus represent a core UPR transcriptome. A heat map that relates this published data set of core ER stress response genes to those that were differentially expressed in the Δ*spfA* mutant is shown in Fig. 3. The first vertical bar on the left highlights genes identified in the core UPR transcriptome (8), and the second vertical bar illustrates their functional classification. The horizontal bars on the right show those ER stress response genes in the core UPR transcriptome that were also differentially expressed in the Δ*spfA* mutant. Despite the fact that *spfA* gene deletion is a very different type of ER stress compared to treatment with agents that cause acute protein unfolding, 47% of the genes that were upregulated by DTT and TM in the core UPR data set were also upregulated in the Δ*spfA* mutant, suggesting that the absence of *spfA* creates an environment of ER stress that necessitates UPR intervention.

Genes that were downregulated in the Δ*spfA* mutant were largely associated with the metabolism of carbohydrates, amino acids, nitrogen, and sulfur (Fig. 2), suggestive of a need for broad metabolic rewiring when *spfA* is absent. In addition, there was downregulation of genes that encode abundantly secreted proteins, which is a type of ER stress response that relieves pressure on the secretory pathway during stress (7). Of particular note was the downregulation of genes involved in the metabolism of lipids, fatty acids, isoprenoids, and inositol phosphate, suggesting a possible dysregulation of membrane homeostasis. In addition, large categories of genes involved in oxidoreductase and electron transport activity were apparent, which could impact the ability to maintain optimal redox balance in the mutant.

### SpfA works in concert with the ER Ca^2+^ pump SrcA to support ER homeostasis.

**FIG 4:**
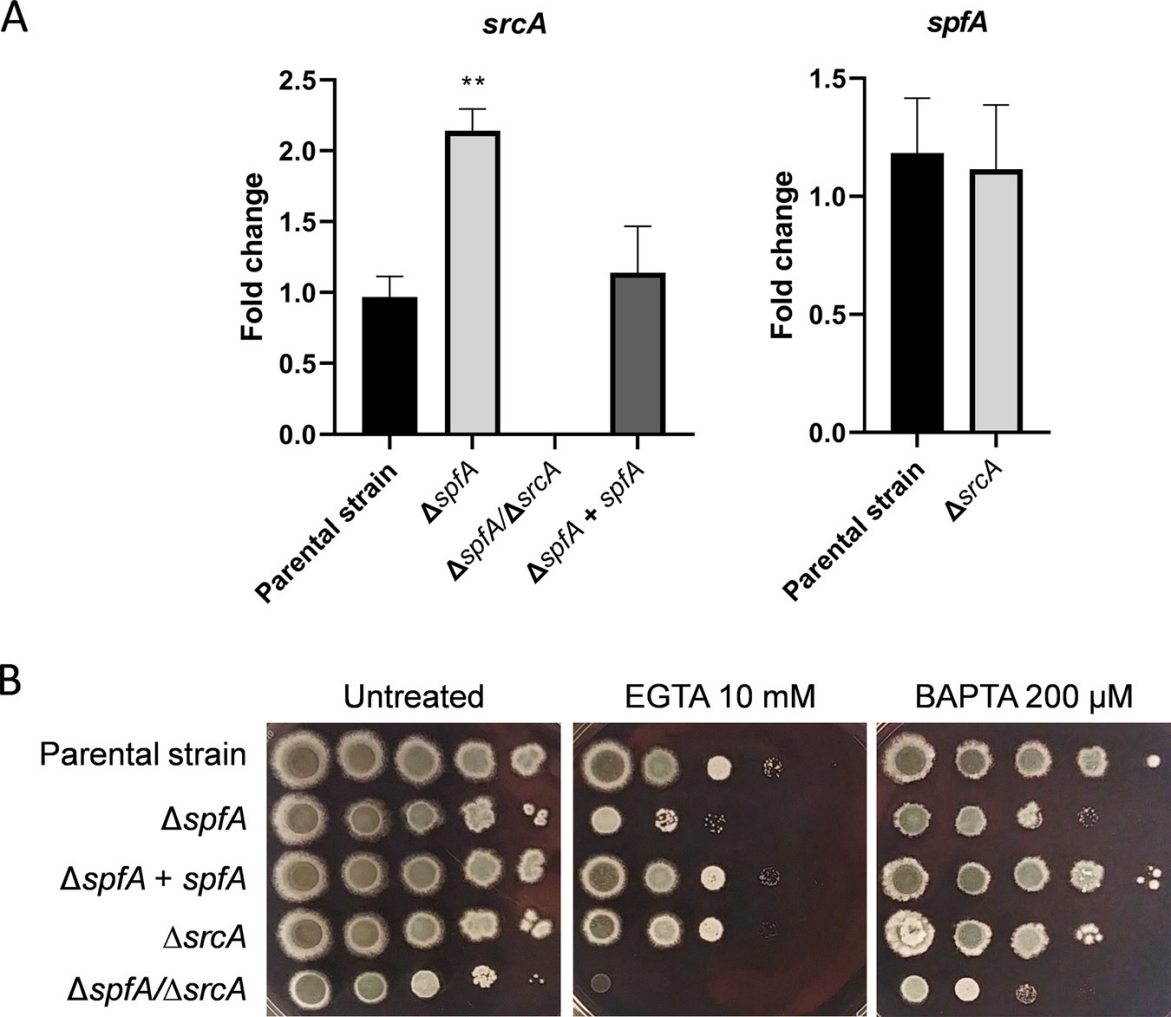
The loss of *spfA* upregulates the expression of the gene encoding the P-type ATPase SrcA and increases susceptibility to cation depletion. (A) RT-qPCR analysis showing the expression of *srcA* and *spfA* mRNAs in each of the indicated strains. The cultures were grown in liquid YG medium for 16 h prior to harvest. Values represent the means ± SD from three technical replicates (**, *P < *0.001 [by one-way ANOVA with Tukey’s *post hoc* test]). (B) Serial 10-fold dilutions of conidia (10^5^ to 10) from the indicated strains were spotted onto AMM plates containing EGTA or BAPTA and incubated for 2 days at 37°C.

We have previously shown that a second P-type ATPase in the ER membrane, SrcA, is upregulated by the UPR during ER stress (18). Since the absence of *spfA* creates an environment of ER stress (Fig. 3), and Ca^2+^ is important to support chaperone function, we hypothesized that *srcA* gene expression would be increased in the Δ*spfA* mutant. Consistent with this, we found increased levels of *srcA* mRNA in the Δ*spfA* mutant by RNA-Seq analysis (Fig. 3, arrow), which was confirmed by RT-qPCR (Fig. 4A). Interestingly, however, no increases in *spfA* levels were observed in the Δ*srcA* mutant. Since Spf1 has also been implicated in cation transport, including Ca^2+^ (27, 28, 32, 33, 43, 44), we hypothesized that A. fumigatus SpfA would work together with SrcA to support cation homeostasis. To test this, a Δ*spfA*/Δ*srcA* double-deletion mutant lacking both of these ATPases was constructed (Fig. S3). Similar to the Δ*spfA* mutant, no difference in conidiation was observed for the Δ*spfA*/Δ*srcA* strain (Fig. S4B), although the double mutant showed a more pronounced radial growth defect (Fig. S4). However, the Δ*spfA* mutant was selectively growth impaired on medium that was rendered cation deficient by supplementation with either EGTA or the more Ca^2+^-specific chelator BAPTA [1,2-bis(2-aminophenoxy)ethane-*N*,*N*,*N*′,*N*′-tetraacetic acid] (Fig. 4B). The growth of the Δ*srcA* mutant was only slightly affected under these conditions, but the Δ*spfA*/Δ*srcA* mutant displayed a more severe growth defect in the presence of either of these chelating agents than the control strains. Taken together, these data provide evidence that SpfA and SrcA jointly support cation homeostasis in the ER.

**FIG 5:**
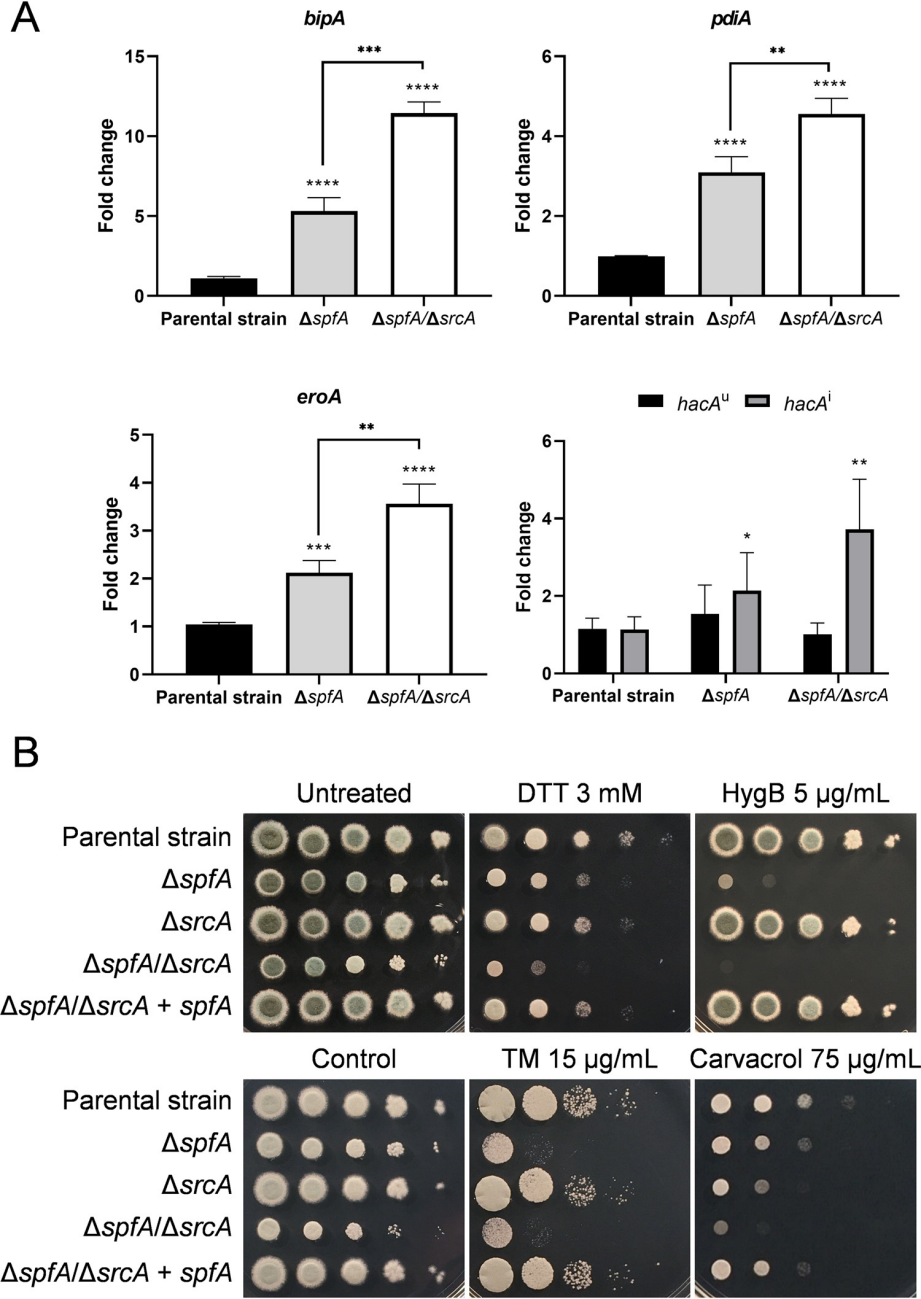
Loss of *spfA* induces the UPR and increases susceptibility to ER stress. (A) Fold change in the level of the induced form of the *hacA* mRNA (*hacA*^i^) as well as the downstream UPR target genes under its control (*bipA*, *pdiA*, and *eroA*) in the indicated strains. The bars represent the means ± SD from three biological replicates per strain and condition (*, *P < *0.05; **, *P < *0.01; ***, *P < *0.001; ****, *P < *0.0001 [by one-way ANOVA with Tukey’s *post hoc* test]). (B) Serial dilutions of conidia (10^5^ to 10) from the indicated strains were incubated for 2 days at 37°C on AMM plates supplemented with the ER stress agent dithiothreitol (DTT), hygromycin B (HygB), tunicamycin (TM), or carvacrol.

Three UPR-dependent genes in the RNA-Seq data set were selected for validation by RT-qPCR analysis, including the Hsp70 chaperone gene *bipA*, the protein disulfide isomerase gene *pdiA*, and the oxidoreductase gene *eroA* (Fig. 3 and Fig. 5A). All three UPR target genes showed elevated expression in the Δ*spfA* mutant (Fig. 5A), with even higher levels of expression evident in the Δ*spfA*/Δ*srcA* mutant. In addition, the loss of *spfA* was associated with increased levels of the *hacA*^i^ mRNA that encodes the bZIP transcription factor HacA that orchestrates the canonical UPR pathway (Fig. 5A). To determine whether the apparent constitutive induction of the UPR in the Δ*spfA* mutant would affect the ability of the fungus to withstand exogenous ER stress, serial dilutions of conidia were spotted onto solid medium supplemented with DTT, TM, or other agents that disrupt ER homeostasis by different mechanisms: loss of translational fidelity by hygromycin B and disruption of ER integrity by carvacrol (45–48). The Δ*spfA* mutant was more susceptible to all four of these ER stress agents (Fig. 5B). The Δ*srcA* mutant also showed marked sensitivity to carvacrol, but the Δ*spfA*/Δ*srcA* mutant revealed even greater susceptibility to DTT, hygromycin B, and carvacrol. In each case, complementation of the deletion of *spfA* in the mutants (Δ*spfA*+*spfA* and Δ*spfA*/Δ*srcA+spfA*, respectively) recapitulated the phenotypes of the parental strain KU80 as well as the Δ*srcA* mutant (Fig. 5B and Fig. S5A and B). Taken together, these findings demonstrate that the loss of *spfA* creates an intracellular environment of increased ER stress, which is exacerbated when *srcA* is also absent, thereby triggering UPR intervention and rendering the fungus susceptible to additional exogenous ER stressors.

10.1128/mBio.02735-21.5 FIG S5 (A and B) Susceptibility of the Δ*spfA* (A) and Δ*srcA* (B) mutants to the indicated ER stress agents is rescued by reconstitution of the genes. Serial dilutions of conidia (10^5^ to 10) from the indicated strains were incubated on AMM plates supplemented with the ER stress agent dithiothreitol (DTT), hygromycin B (HygB), tunicamycin (TM), or carvacrol for 2 days at 37°C. (C and D) Comparison of the susceptibilities of the Δ*spfA* mutant to the indicated stress agents in YPD medium and YG medium, respectively. Download FIG S5, JPG file, 1.7 MB. Copyright © 2021 Guirao-Abad et al. 2021 Guirao-Abad et al. https://creativecommons.org/licenses/by/4.0/ This content is distributed under the terms of the Creative Commons Attribution 4.0 International license.

### SpfA supports membrane homeostasis in A. fumigatus.

**FIG 6:**
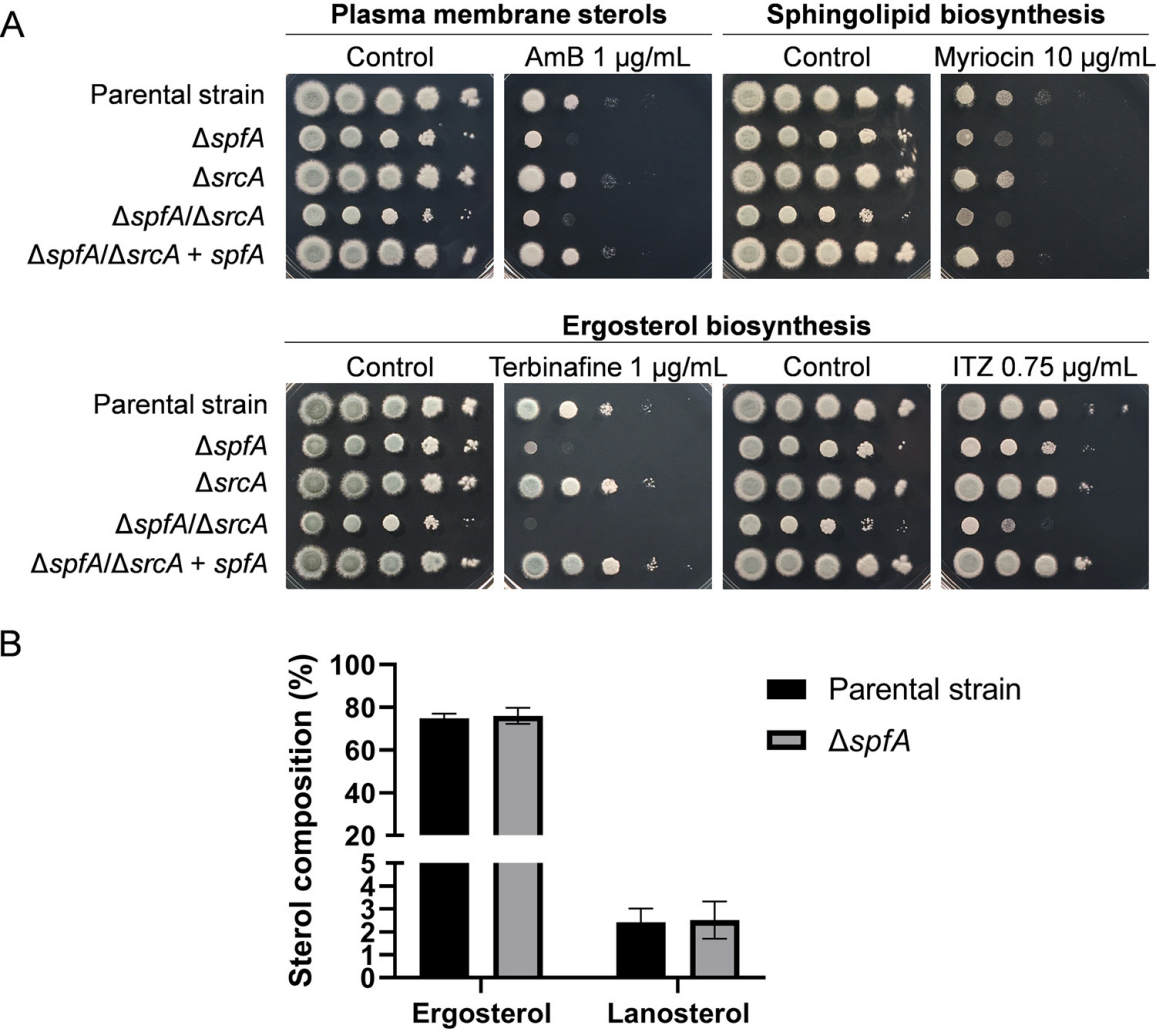
The *spfA* gene confers resistance to agents that target membrane homeostasis. (A) Serial dilutions of conidia (10^5^ to 10) from the indicated strains were incubated for 2 days at 37°C on AMM plates supplemented with amphotericin B (AmB), myriocin, terbinafine, or itraconazole (ITZ). Comparable findings were observed in rich medium (see Fig. S5C and D in the supplemental material). (B) Analysis of the sterol composition of the Δ*spfA* mutant, as described in Materials and Methods.

The downregulation of genes involved in the metabolism of lipids, fatty acids, isoprenoids, and inositol phosphate in the Δ*spfA* mutant (Fig. 2) suggested that the ability to maintain membrane homeostasis might be impaired in the absence of SpfA. To test this, mutants lacking *spfA* were exposed to antifungal drugs that target two major types of membrane lipids, ergosterol and sphingolipids. The Δ*spfA* mutant exhibited greater susceptibility to amphotericin B (AmB), a member of the polyene class that disrupts membrane integrity by interacting with ergosterol, as well as terbinafine, an inhibitor of squalene epoxidase in the ergosterol biosynthetic pathway (Fig. 6A). A previous study showed that the S. cerevisiae Δ*spf1* mutant is very susceptible to myriocin, which disrupts sphingolipid synthesis (25). However, the Δ*spfA* mutant showed only a slight increase in susceptibility to myriocin as well as itraconazole, which blocks ergosterol biosynthesis by inhibiting lanosterol C-14 demethylase (at a later step than terbinafine). While the Δ*srcA* mutant revealed wild type-like susceptibility to all four of these compounds, the Δ*spfA*/Δ*srcA* mutant showed an increase in susceptibility to AmB that was comparable to that of the Δ*spfA* mutant, slightly increased susceptibility to terbinafine and myriocin but a more pronounced growth defect in the presence of itraconazole (Fig. 6A). The Δ*spfA* mutant showed similar levels of susceptibility to these compounds in either yeast extract-peptone-dextrose (YPD) or YG medium (Fig. S5C and D). Drug susceptibility testing by gradient diffusion MIC analysis revealed a similar increase in susceptibility to AmB in the Δ*spfA* mutant but no apparent increase in susceptibility to itraconazole or voriconazole unless *srcA* was also absent (Fig. S6). Together, these results demonstrate that the loss of *spfA* increases susceptibility to agents that target membrane sterols, possibly due to the dysregulation of genes involved in membrane homeostasis observed in this mutant (Fig. 2). Since the susceptibility to AmB and terbinafine was *spfA* dependent, we compared the sterol profile of the Δ*spfA* mutant of A. fumigatus to that of its parental strain. However, in contrast to yeast, which showed a 4-fold increase in the ergosterol-to-lanosterol ratio in the absence of *spf1* (25), this sterol ratio was unaltered in the Δ*spfA* mutant (Fig. 6B).

10.1128/mBio.02735-21.6 FIG S6 Analysis of antifungal drug susceptibility by gradient diffusion. Conidia from the indicated strains (10^6^ conidia/ml) were spread with a cotton swab onto the surface of a plate of either RPMI 1640 agar medium containing 0.164 M morpholino propanesulfonic acid (MOPS) and l-glutamine (pH 7.0) (A) or AMM (B). Once dried, the surface was overlaid with an MIC test strip (Liofilchem) containing a gradient of the indicated drugs: caspofungin (CSF), amphotericin B (AmB), itraconazole (ITZ), and voriconazole (VOR). Plates were incubated for 2 days at 37°C. Download FIG S6, JPG file, 2.8 MB. Copyright © 2021 Guirao-Abad et al. 2021 Guirao-Abad et al. https://creativecommons.org/licenses/by/4.0/ This content is distributed under the terms of the Creative Commons Attribution 4.0 International license.

### SpfA and SrcA jointly support redox homeostasis.

**FIG 7:**
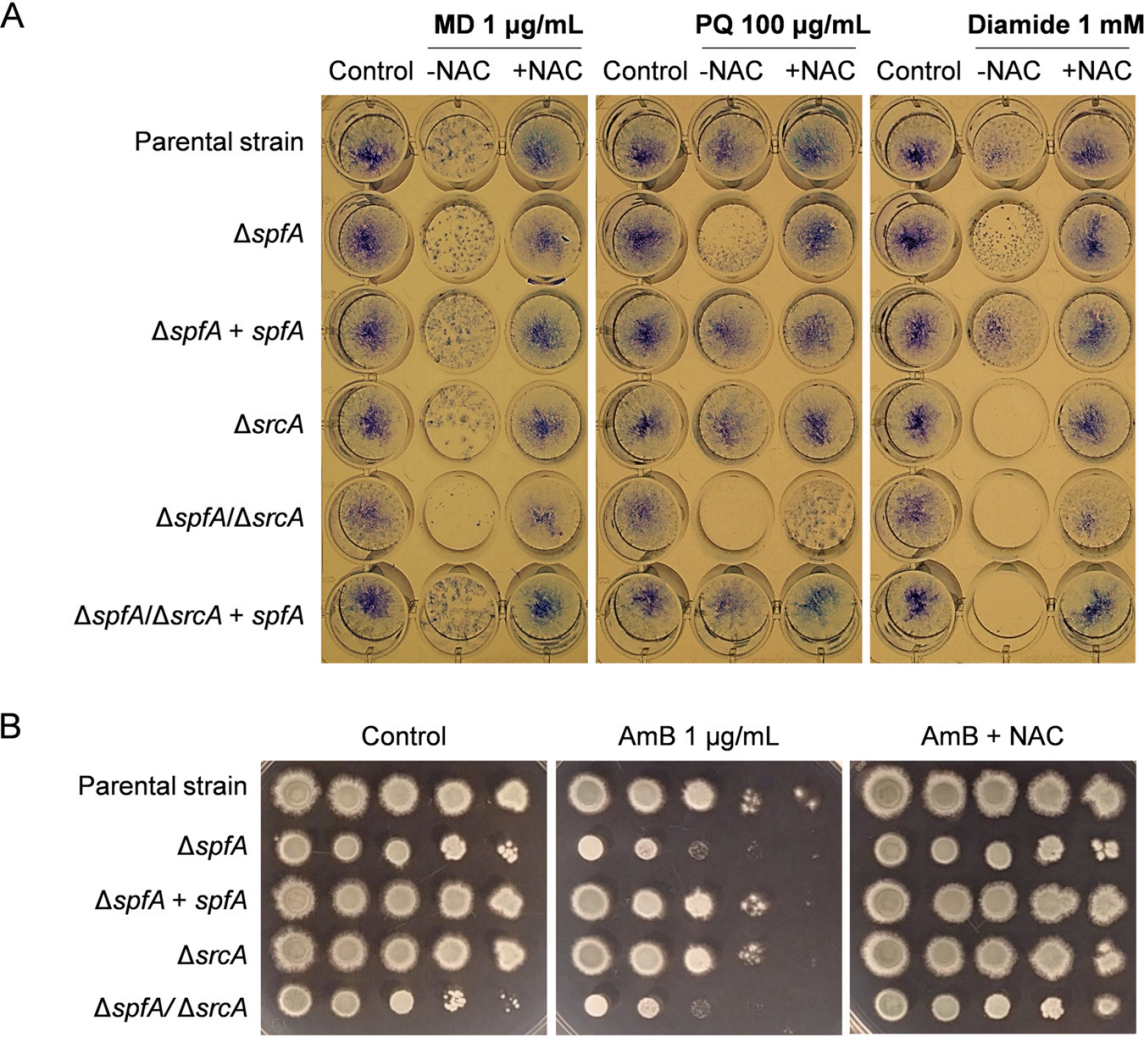
Loss of *spfA* increases susceptibility to oxidative stress. (A) Conidia were inoculated into multiwell plates of liquid AMM containing the indicated concentrations of the oxidative stress-inducing agent menadione (MD), paraquat (PQ), or diamide in the presence or absence of 5 mM the antioxidant *N*-acetyl-cysteine (NAC). Multiwell plates were incubated for 24 h at 37°C. The biomass attached to the bottom of the wells was highlighted by staining with methylene blue prior to photography. (B) Susceptibility to amphotericin B (AmB) is reversed by supplementation with NAC. Serial dilutions of conidia (10^5^ to 10) from the indicated strains were incubated for 2 days at 37°C on AMM plates supplemented with AmB in the presence or absence of NAC.

Since an alteration in the redox environment of the cell can be detrimental to the structure and function of proteins (49), the reduced levels of oxidoreductase and electron transport activity in the Δ*spfA* mutant (Fig. 2) raised the possibility that the loss of *spfA* could increase the vulnerability of the fungus to further oxidative stress. To test this, strains were grown in multiwell plates containing liquid medium supplemented with agents that generate reactive oxygen species (ROS), and the resulting biomass at the bottom of the wells after 24 h of growth was stained with methylene blue to enhance visibility. The Δ*spfA* mutant showed a slight reduction in biomass formation in the presence of the ROS-generating agent menadione (MD) or paraquat (PQ), which was more apparent in the Δ*spfA*/Δ*srcA* mutant (Fig. 7A). Surprisingly, the thiol-oxidizing agent diamide had only a mild inhibitory effect on the Δ*spfA* mutant but completely inhibited the growth of the Δ*srcA* and double mutants. Oxidative stress has also been implicated in the toxicity of AmB toward pathogenic fungi (50). Interestingly, the increased susceptibility of the Δ*spfA* mutant to AmB could be rescued by the ROS scavenger *N*-acetyl-l-cysteine (NAC) supplemented into solid medium, suggesting that the reduced capacity of the Δ*spfA* mutant to withstand oxidative stress contributes to the susceptibility of this mutant to AmB (Fig. 7B).

### SpfA provides resistance to cell wall stress.

**FIG 8:**
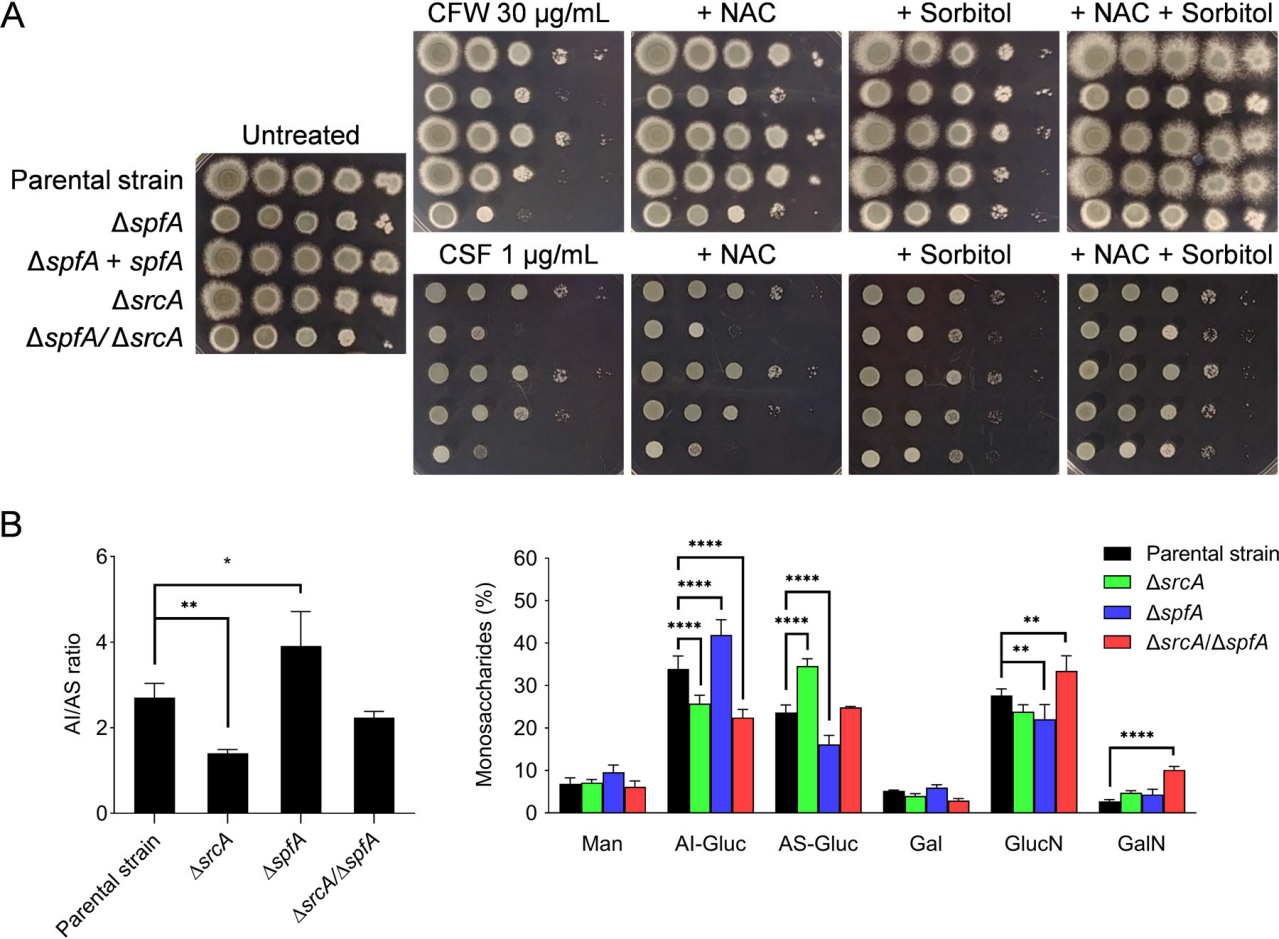
Loss of *spfA* alters cell wall composition and increases susceptibility to cell wall stress. (A) Serial 10-fold dilutions of conidia from the indicated strains were spotted onto AMM plates containing calcofluor white (CFW) or caspofungin (CSF) in the presence or absence of 5 mM NAC and/or 0.8 M sorbitol. The plates were incubated for 2 days at 37°C. (B) Biochemical analysis of the cell wall. (Left) Ratio of the alkali-insoluble (AI) fraction to the alkali-soluble (AS) fraction of the indicated strains. (Right) Percent monosaccharide composition of the cell wall in the same strains. Man, mannose; Gluc, glucose; Gal, galactose; GlucN, glucosamine; GalN, galactosamine.

Both the Δ*spfA* and the Δ*srcA* mutants revealed increased susceptibility to the cell wall-damaging agent calcofluor white (CFW), which was accentuated by the absence of both genes (Fig. 8A). This phenotype was partially corrected by the addition of nontoxic levels of either NAC or the osmotic stabilizer sorbitol (Fig. 8A and Fig. S7), consistent with evidence that both oxidative stress and the loss of cell wall integrity contribute to the toxicity of this compound (50). The antifungal drug caspofungin (CSF) weakens the cell wall by impairing β-glucan synthase activity (51), and the Δ*spfA* mutant demonstrated increased susceptibility to this compound in either minimal medium, RPMI 1640, or YG (Fig. 8, Fig. S5, and Fig. S6). However, the Δ*spfA*/Δ*srcA* mutant showed no further increase in CSF susceptibility relative to the Δ*spfA* mutant either on spot dilution plates (Fig. 8A) or by gradient diffusion MIC analysis (Fig. S6), indicating that the absence of *spfA* is responsible for this cell wall phenotype. However, in contrast to CFW, sorbitol was much more effective than NAC in rescuing the Δ*spfA* mutant from CSF toxicity, suggesting that a loss of cell wall integrity rather than impaired oxidative stress resistance was responsible for CSF hypersensitivity. Findings comparable to the CFW results were made using the cell wall stress agent Congo red (CR), to which the Δ*spfA* mutant showed increased susceptibility that could be rescued by osmotic stabilization of the medium (Fig. S7).

10.1128/mBio.02735-21.7 FIG S7 (A) Lack of toxicity of NAC and sorbitol at concentrations used to reverse oxidative and cell wall stress (Fig. 7 and 8). Serial 10-fold dilutions of conidia from the indicated strains were spotted onto AMM plates containing 5 mM NAC or 0.8 M sorbitol. (B) Loss of *spfA* increases sensitivity to CR. Serial 10-fold dilutions of conidia from the indicated strains were spotted onto AMM plates containing 20 μg/ml Congo red in the presence or absence of NAC and/or sorbitol. Plates were incubated for 2 days at 37°C. Download FIG S7, JPG file, 2.1 MB. Copyright © 2021 Guirao-Abad et al. 2021 Guirao-Abad et al. https://creativecommons.org/licenses/by/4.0/ This content is distributed under the terms of the Creative Commons Attribution 4.0 International license.

The susceptibility of the Δ*spfA* mutant to CFW, CSF, and CR suggested that the loss of *spfA* adversely affects diverse aspects of cell wall integrity. The A. fumigatus hyphal cell wall is biochemically divided into an alkali-insoluble (AI) fibrillar “skeleton,” which is mostly composed of β-(1-3)-glucan, chitin, and galactomannan, and an alkali-soluble (AS) “cement” predominantly containing α-(1-3)-glucan, galactosaminogalactan, and galactomannan (52, 53). As recently reported (18), a decrease in the AI/AS ratio was observed for the Δ*srcA* mutant relative to its parental strain KU80 (Fig. 8B). In contrast, the Δ*spfA* mutant displayed a significantly higher AI/AS ratio, which could be attributed to a higher percentage of glucose in the AI fraction (Fig. 8B), suggesting increased biosynthesis of β-(1,3)-glucan to support the cell wall. This could be due to SrcA since the Δ*srcA* mutant showed significantly decreased β-(1,3)-glucan in the cell wall compared to the parental strain, while *srcA* mRNA was upregulated in the Δ*spfA* mutant. On the other hand, the Δ*spfA*/Δ*srcA* mutant showed significantly decreased cell wall β-(1,3)-glucan but a higher percentage of the chitin-forming monomer glucosamine in its cell wall, indicative of a compensatory response to the decreased β-(1,3)-glucan content. Taken together, these results demonstrate that SpfA and SrcA cooperatively influence the composition and integrity of the cell wall of A. fumigatus.

### SpfA and SrcA jointly support the virulence of A. fumigatus.

**FIG 9:**
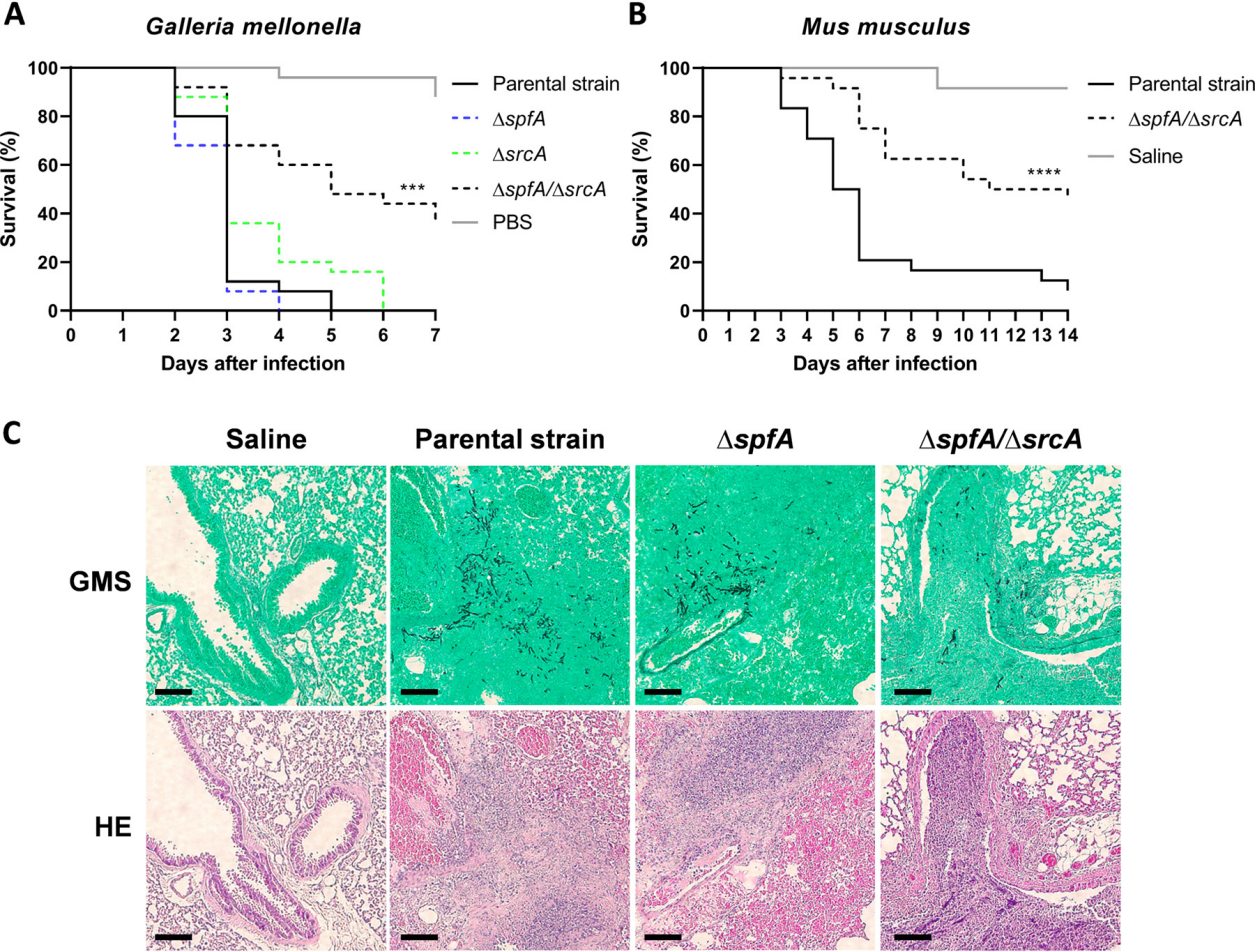
SpfA and SrcA jointly contribute to virulence. (A and B) Percent survival over time following infection with conidia from the indicated fungal strains in an immunocompetent insect model (G. mellonella) and an immunosuppressed mouse model (Mus musculus). The graph shows pooled data from male and female mice (***, *P < *0.001; ****, *P < *0.0001 [by a log rank test]. (C) Histopathological analysis of lungs from mice treated as described above for panel B and sacrificed 3 days after infection. Tissue sections were stained with Gomori’s methenamine silver (GMS) or hematoxylin and eosin (HE). Bars, 100 μm.

Since the *spfA* gene is a downstream target of the canonical UPR (Fig. 1), and this stress response pathway is a known regulator of fungal pathogenesis (54), we assessed the contribution of *spfA* to virulence. Using larvae of the greater wax moth, Galleria mellonella, as an immunocompetent animal infection model, we found that the Δ*spfA* mutant retained full virulence, but the Δ*spfA*/Δ*srcA* mutant showed a reduced capacity to induce mortality (Fig. 9A). Similar findings of attenuated virulence in the Δ*spfA*/Δ*srcA* mutant, but not the Δ*spfA* mutant, were demonstrated using an immunosuppressed mouse model of pulmonary aspergillosis, using both male and female mice (Fig. 9B and Fig. S8). Histopathological analysis on day 3 postinfection revealed comparable levels of fungal growth and surrounding inflammation in lung tissue derived from mice infected with the control strain of A. fumigatus and mice infected with the Δ*spfA* mutant. However, the extent of fungal growth in the Δ*spfA*/Δ*srcA* mutant was decreased relative to that of its parental strain, with less evidence of invasion into the lung parenchyma (Fig. 9C). These observations indicate that the ER-resident P-type ATPases SpfA and SrcA cooperate during fungal pathogenicity.

10.1128/mBio.02735-21.8 FIG S8 SpfA and SrcA jointly contribute to virulence in male and female mice. (A) Percent survival over time following infection with conidia from the indicated fungal strains in immunosuppressed male and female mice. Data for the comparison between the parental strain and the Δ*spfA*/Δ*srcA* mutant are the same as the pooled data shown in Fig. 9, with the mice separated by sex. *, *P < *0.05; **, *P < *0.01 (by a log rank test). (B) The same inoculum of conidia used to inoculate the mice in panel A was spotted onto the surface of lung explants from uninfected mice and incubated for 24 h at 37°C. Download FIG S8, JPG file, 1.7 MB. Copyright © 2021 Guirao-Abad et al. 2021 Guirao-Abad et al. https://creativecommons.org/licenses/by/4.0/ This content is distributed under the terms of the Creative Commons Attribution 4.0 International license.

## DISCUSSION

Spf1 is a P5-type ATPase in the ER membrane that has recently been implicated in a novel ER quality control mechanism that maintains ER homeostasis by selectively extracting transmembrane proteins that are mistargeted to the ER (31). This provides evidence that polypeptides represent a new class of P-type ATPase substrate, which is consistent with the unusually large substrate-binding pocket of Spf1 relative to the structure of other P-type ATPases that transport ions and lipids (31). In this study, we assessed the contribution of the Spf1 ortholog in A. fumigatus, termed SpfA, to the biology and virulence of the fungus.

Whole-transcriptome sequence analysis revealed that the loss of the A. fumigatus *spfA* gene differentially regulated the expression of more than 1,300 genes, involving approximately 13% of the open reading frames in this fungal species (Fig. 2A). Genes that comprise the UPR or other aspects of ER quality control were prominent in one of the two major categories of upregulated genes (Fig. 2B), many of which overlap a previously published data set of core UPR genes that are induced under conditions of acute ER stress (Fig. 3) (8). This implies that the loss of *spfA* disrupts ER homeostasis to an extent similar to that with powerful ER stress-inducing agents, necessitating the activation of the canonical UPR (Fig. 5A). This upregulation of UPR target genes was also associated with an overall increase in mRNAs involved in the translational machinery (Fig. 2B). Since ribosome biogenesis and protein translation are energetically costly to a cell, increasing their activity under conditions of acute stress suggests that there is an urgent need for the expression of homeostatic proteins, including those that show increased abundance as a consequence of UPR activation. We speculate that the increased susceptibility of the Δ*spfA* mutant to hygromycin B (Fig. 5B) is due, at least in part, to the ability of this aminoglycoside to impair ribosome function and thus reduce the effectiveness of the upregulation of the translational machinery. The precise mechanism by which the loss of *spfA* causes ER stress is likely to be pleiotropic. For example, recent evidence demonstrating ER transmembrane dislocase activity for Spf1 suggests that the loss of Spf1 would cause a backup of mislocalized proteins in the ER membrane, thereby contributing to ER dysfunction. Consistent with this, we found that the Δ*spfA* mutant exhibited increased susceptibility to ER stress agents in both minimal and rich media (Fig. 5B and Fig. 6B), indicating that the absence of *spfA* brings the baseline level of ER stress within the fungus closer to the maximum tolerable level.

In addition to increased ER stress susceptibility, the Δ*spfA* mutant was more susceptible to agents that deplete extracellular cations or confer oxidative stress (Fig. 4B, Fig. 6B, and Fig. 7). The Δ*spfA*/Δ*srcA* mutant showed greater vulnerability to these agents than either of the single mutants, suggesting redundancy between these two P-type ATPases in supporting the redox and cationic environment of the fungus. The Δ*spfA* mutant also showed increased susceptibility to antifungal drugs, including AmB, terbinafine, and caspofungin (Fig. 6; see also Fig. S6 in the supplemental material). AmB susceptibility was almost completely rescued by the addition of the antioxidant NAC, indicating that a substantial amount of the toxicity of AmB toward the Δ*spfA* mutant is due to oxidative stress (Fig. 7B), which is a known effect of this antifungal drug (50). The Δ*spfA* and Δ*spfA*/Δ*srcA* mutants showed similar levels of increased susceptibility to the ergosterol biosynthesis inhibitor terbinafine, demonstrating that it is the loss of *spfA* alone that confers this phenotype. We speculate that the greater susceptibility of the Δ*spfA* mutant to terbinafine is due to the downregulation of genes involved in lipid, fatty acid, isoprenoid, and inositol phosphate metabolism in this mutant (Fig. 2B), making it more difficult for the fungus to compensate for a loss of membrane homeostasis caused by blocking an early step in the ergosterol biosynthetic pathway. The *spf1* deletion mutant of C. albicans displays a strong increase in susceptibility to fluconazole (32). Although A. fumigatus is intrinsically resistant to fluconazole, it is susceptible to itraconazole (55), which acts at the same step as fluconazole. However, the susceptibility of A. fumigatus Δ*spfA* to itraconazole was either not detectable (Fig. S6) or only slightly increased relative to the wild type (Fig. 6A and Fig. S5C), indicating a fundamental difference between these two fungal species (32). Since S. cerevisiae lacks a SERCA-type Ca^2+^ ATPase (56), and clear orthologs are also not apparent in the C. albicans or Candida glabrata genome, it is interesting to speculate that their presence in A. fumigatus reflects a level of functional redundancy between SpfA and SrcA homologs that evolved to meet the unique environmental pressures that this mold encounters in its ecological niche.

The Δ*spfA* mutant demonstrated increased susceptibility to a number of cell wall stress agents (Fig. 8A), suggesting that it harbors a defect in cell wall integrity. This was confirmed, revealing an increase in β-(1,3)-glucan in the Δ*spfA* mutant, a decrease in α-(1,3)-glucan in the Δ*srcA* mutant, and an increase in chitin in the Δ*spfA*/Δ*srcA* mutant (Fig. 8B). Since these three components represent the major cell wall polysaccharides of A. fumigatus, and the cross-linking of β-(1,3)-glucan and chitin is important for the strength of the fibrillar skeleton, these alterations provide evidence that compensatory changes in cell wall composition are activated in an attempt to offset a weakened cell wall caused by the loss of *spfA* and/or *srcA*.

The Δ*spfA* and Δ*srcA* mutants displayed virulence properties similar to those of the parental strain, but the Δ*spfA*/Δ*srcA* strain was attenuated in both a *Galleria* insect model and an immunosuppressed mouse model of fungal infection. This demonstrates that the substrates transported by SrcA and SpfA are individually dispensable for virulence but are collectively necessary to support the adaptation of the fungus to the adverse environment of the host. Unlike the UPR-deficient Δ*hacA* mutant, which grows poorly on explants of mouse lung tissue due to reduced secretory capacity (9), the growth of the Δ*spfA*, Δ*srcA*, and Δ*spfA*/Δ*srcA* mutants on lung explants was comparable to their behavior on standard laboratory medium (Fig. S8), suggesting that the reduced virulence of the Δ*spfA*/Δ*srcA* mutant is not a consequence of an inability to secrete hydrolytic enzymes that are needed to extract nutrients from tissues. It is likely that the attenuated virulence of the Δ*spfA*/Δ*srcA* mutant can be partly attributed to its reduced growth rate (Fig. S4). However, it should be noted that the growth rate of this mutant on rich medium is four times higher than that of a previously reported Δ*srcA*/Δ*pmrA* mutant, yet both mutants show comparable levels of attenuated virulence (18), suggesting that the reduced ability of the Δ*spfA*/Δ*srcA* mutant to infect a host involves a lack of fitness *in vivo* that extends beyond growth rate.

Taken together, the findings in this study add *spfA* to the list of genes encoding P-type ATPases that are downstream of the UPR in A. fumigatus and that collectively support the ability of this organism to adapt to stress, including the adverse pressures exerted by the host during infection. The ability of *spfA* and *srcA* and to impinge on similar phenotypes suggests that their encoded proteins buffer one another such that the absence of one can be at least partially compensated for by the other. This implies a level of genetic interaction, particularly with respect to Ca^2+^ homeostasis, the oxidative stress response, ER stress tolerance, and virulence, and is consistent with our finding that that loss of SpfA is associated with increased levels of srcA (Fig. 4). Since the UPR regulates virulence-related traits in diverse species of human- and plant-pathogenic fungi (8, 9, 11–17), further analysis of the mechanisms involved in UPR regulation has the potential to uncover novel targets for therapeutic intervention.

## MATERIALS AND METHODS

### Strains and growth conditions.

The strains of A. fumigatus used throughout this study are listed in Table S1 in the supplemental material. Unless otherwise stated, Aspergillus minimal medium (AMM) (1% [wt/vol] d-glucose, 1% [vol/vol] NH_4_ tartrate, 2% [vol/vol] salt solution [2.6% {wt/vol} KCl, 2.6% {wt/vol} MgSO_4_ heptahydrate, 7.6% {wt/vol} KH_2_PO_4_, 5% {vol/vol} trace element solution]) was used for all stress tests to optimize the reproducibility of growth phenotypes, adding 0.8% (wt/vol) UltraPure agarose (Invitrogen) when growth on solid medium was required. Conidia were harvested from mycelia grown for 1 week at 37°C on OSM (osmotically stabilized medium) composed of solid AMM supplemented with 1.2 M sorbitol. Radial growth measurements were obtained by spotting 5 × 10^3^ conidia in a 5-μl droplet onto the center of YG (0.5% yeast extract, 2% glucose) or IMA (inhibitory mold agar; Becton, Dickinson) plates and monitoring the colony diameter over time. Stress sensitivities were determined by spotting serial 10-fold dilutions of conidia (from 10^5^ to 10 spores in droplets of 5 μl) onto AMM plates containing the compound to be tested. Chemicals used to induce stress include BAPTA (Invitrogen), EGTA (Fisher), dithiothreitol (Thermo Scientific), tunicamycin (Cayman Chemical), hygromycin B (RPI), carvacrol (Sigma), amphotericin B (Cayman Chemical), myriocin (Sigma), terbinafine (Sigma), itraconazole (Sigma), calcofluor white (Sigma), caspofungin (Cayman Chemical), and Congo red (Sigma). Susceptibility to oxidative stress was assessed by growing 10^3^ conidia in 24-well plates containing 2 ml of liquid AMM with the oxidative agent menadione (Sigma), paraquat (Acros Organics), or diamide (Sigma). The antioxidant compound *N*-acetyl-l-cysteine (NAC; Sigma) was added where indicated. For agents that were solubilized in dimethyl sulfoxide (DMSO) (amphotericin B, tunicamycin, carvacrol, itraconazole, and myriocin) or acetone (terbinafine), control plates contained the same concentration of the vehicle that was present in the test plates, which was ≤0.1% of the total volume. At this concentration of the vehicle, no adverse effects on growth were observed on control plates. For qualitative evaluation of growth in liquid culture, mycelia were grown for 48 h at 37°C, and the biomass obtained was stained with 0.5% (wt/vol) methylene blue (Fisher) for 1 h at room temperature, washed with water, and then completely dried prior to photography. For analysis of antifungal drug susceptibility by gradient diffusion, conidia (10^6^ conidia/ml) were spread with a cotton swab onto the surface of a plate of RPMI 1640 agar medium containing 0.164 M morpholino propanesulfonic acid (MOPS) and l-glutamine (pH 7.0). Once dried, the surface was overlaid with an MIC test strip (Liofilchem) containing a gradient of caspofungin, amphotericin B, itraconazole, or voriconazole, and the plates were incubated for 2 days at 37°C.

10.1128/mBio.02735-21.9 TABLE S1 Strains of Aspergillus fumigatus used in this study. Download Table S1, DOCX file, 0.03 MB. Copyright © 2021 Guirao-Abad et al. 2021 Guirao-Abad et al. https://creativecommons.org/licenses/by/4.0/ This content is distributed under the terms of the Creative Commons Attribution 4.0 International license.

### Genetic modifications.

Gene deletion, mutant complementation, and *in situ* tagging were achieved by homologous recombination with the target locus using 5′- and 3′-flanking regions of about 1 kb in size that were PCR amplified from genomic DNA of the parental strain KU80 (399) (Table S1). Recipient strains for transformations contained a deletion of the *akuB*^KU80^ gene for efficient site-specific integration as previously described (57). A recyclable marker module (MM) was used for selection (58), which contained the chlorimuron-ethyl resistance (*cme^R^*) gene as well as the beta-recombinase (β-*rec*) gene under the control of a xylose-responsive promoter (P*xyl*), which was flanked by two *six* sites for β-Rec-mediated self-excision of the MM. The MM was PCR amplified with primer pair 1053/1054 (Table S2) from the vector p680 (*cme^R^*-β-*rec*; gift from Jean-Paul Latgé). The pUC19L backbone for selection and cloning in bacteria was PCR amplified from vector pUC19 with primer pair 1061/1062 and contained restriction enzyme recognition sites for linearization prior to transformation into the fungus.

10.1128/mBio.02735-21.10 TABLE S2 List of oligonucleotides used in this study. Download Table S2, DOCX file, 0.02 MB. Copyright © 2021 Guirao-Abad et al. 2021 Guirao-Abad et al. https://creativecommons.org/licenses/by/4.0/ This content is distributed under the terms of the Creative Commons Attribution 4.0 International license.

For *in situ* tagging of the *spfA* gene (Afu3g13790) with the *mrfp1* (monomeric red fluorescent protein 1) coding sequence, the left arm spanning a 3′ portion of the *spfA* gene was PCR amplified with primer pair 1434/1435, the right arm (the *spfA* 3′ region downstream of the stop codon) was amplified with primer pair 1436/1437, and the *mrfp1* gene was amplified from genomic DNA of an mRFP1-H2A-expressing strain (gift from Tobias Hohl) (59) with primer pair 1438/1439. The fragments were assembled with *cme^R^*-β-*rec* and pUC19L using the GeneArt seamless cloning and assembly kit (Thermo Fisher), creating plasmid p715. Transformation of the fungus was performed as described previously (60), using 6 μg of linearized plasmid DNA to transform protoplasts of mycelia grown in YG medium for 17 h at 30°C at 140 rpm (61). The ScaI-linearized p715 plasmid was transformed into the SrcA-eGFP-expressing recipient strain 725 (18). For selection, transformants were plated directly onto OSM plates supplemented with 50 μg/ml chlorimuron ethyl (Fisher Scientific). Monoconidial transformants were passaged onto AMM plates containing 1% (wt/vol) xylose as the sole carbon source to excise the MM. The site-specific integration of the *spfA-mrfp1* construct was verified by PCR (data not shown). The coexpression of both SpfA-mRFP1 and SrcA-eGFP was confirmed by epifluorescence microscopy (Fig. 1A).

To create an *spfA* gene knockout construct, the left and right arms flanking the *spfA* open reading frame were PCR amplified with primer pairs 1386/1387 and 1388/1389, respectively, and assembled with the *cme^R^*-β-*rec* and pUC19L fragments mentioned above, resulting in vector p706. To obtain a Δ*spfA* mutant strain (772), cultures of the parental strain KU80 (399) were transformed with the FspI-linearized p706 plasmid. In the same manner, the Δ*spfA*/Δ*srcA* double mutant (756) was created by transforming a Δ*srcA* strain (402) with FspI-linearized p706. Confirmation of all genotypes was performed by PCR (Fig. S3 and Table S2).

For complementation of the Δ*spfA* deletion mutants, the *spfA* open reading frame, including 5′- and 3′-flanking regions of 1 kb each, was first PCR amplified from KU80 using primer pair 1432/1433. Next, a pUC19L vector backbone containing a 1.9-kb intergenic region (IR) from chromosome 1 (between the loci Afu1g04960 and Afu1g04970) for site-specific genomic integration was PCR amplified from plasmid p675 (18) with the primer pair 1309/1310. Next, the 6.3-kb *spfA* amplicon and the 4.5-kb pUC19L-IR fragment were ligated using PacI and AscI restriction sites, resulting in the complementation plasmid p713. To complement the Δ*spfA* (772) and Δ*spfA*/Δ*srcA* (756) mutants, protoplasts were respectively cotransformed with BsaBI-linearized p713 and the selectable marker vector p680 (linearized with FspI) in a stochiometric ratio of 10 to 1. Confirmation of complemented genotypes was performed by PCR analysis (Fig. S3).

### Bright-field and fluorescence microscopy.

Approximately 1 × 10^3^ conidia of strain 826 expressing SpfA-mRFP1 and SrcA-eGFP (Table S1) were first pipetted onto glass coverslips in petri dishes filled with liquid AMM and incubated for 17 h at 30°C without agitation. For live-cell imaging, the samples were then mounted onto glass carriers and analyzed using an Olympus BX51 microscope equipped with a 100×/1.35 oil immersion objective. Bright-field and fluorescence images of germlings were captured with a Diagnostic Instruments RTKE fluorescence imaging camera adjusted for brightness, contrast, and color with the open-source program Fiji (https://fiji.sc/).

### Real-time quantitative PCR analysis.

Cultures were grown in YG medium to facilitate comparisons with a previously published data set of ER stress response genes that was obtained using this medium (8). Cultures containing 50 ml of liquid YG medium were inoculated with 1 × 10^6^ conidia/ml and incubated for 16 h at 37°C at 200 rpm. The resultant biomass was ground under liquid nitrogen, and the RNA was extracted using an RNAzol RT column kit (MRC, Inc.). The purified RNA was treated with DNase I (Roche), and the cDNA was synthesized using the iScript reverse transcription supermix for RT-qPCR (Bio-Rad). To quantify gene expression, a mix with 1 μg of cDNA, 500 nM the gene-specific primers listed in Table S2 (200 nM for the 18S rRNA housekeeping gene), and the iTaq universal SYBR green supermix (Bio-Rad) was made. Each reaction was run in triplicates in a StepOne real-time PCR system (Applied Biosystems). The amplification parameters were set to 20 s at 95°C, 40 cycles of 3 s at 95°C, and 30 s at 60°C (with the exception of 20 s at 66°C for *hacA*^u/i^ primers). Melting curves were generated to verify the specificity of the reactions, and the primer efficiencies (between 95 and 105%) were determined with cDNA standard curves. The results are presented as fold changes in transcript levels in comparison to samples from the parental strain or untreated controls.

### RNA sequencing and analysis.

Cultures were grown in YG medium to facilitate comparisons with a previously published data set of ER stress response genes that was obtained using this medium (8). A Direct-zol RNA MiniPrep kit (Zymo Research) was used for RNA isolation, and RNA integrity was confirmed using an Agilent 2100 Bioanalyzer instrument prior to submission for RNA sequencing (data not shown). Library preparation was performed using the NEBNext Ultra II directional RNA library prep kit for RNA-Seq. Poly(A) RNA-Seq was carried out in the Genomics, Epigenomics, and Sequencing Core (GESC) at the University of Cincinnati using a NextSeq 550 system (Illumina). After adapter trimming and quality control, fastq files of each sample were merged before data analysis. Gene expression analysis was carried out using the A. fumigatus Af293 reference genome and annotation tracks from FungiDB (release 51) in CLC Genomics Workbench (Qiagen). Expression values were estimated as transcripts per million (TPM). Differential expression for RNA-Seq was calculated between the control and mutant strains using TMM (trimmed mean of M values) as the normalization method. Downstream analysis was performed using FungiDB (62) and FungiFun2 (39).

### Analysis of cell wall monosaccharide composition.

Cultures were grown in YG medium to facilitate a comparison with the RNA-Seq data generated in this study. Cell wall analysis was performed as previously described (9). Flasks containing 50 ml of liquid YG medium were inoculated with 1 × 10^8^ conidia and incubated for 24 h at 37°C under constant shaking (150 rpm). Mycelia were collected by filtration and subjected to cell wall carbohydrate analysis as previously described (7). The composition of the monosaccharides identified in the alkali-soluble (AS) and alkali-insoluble (AI) fractions was calculated for each strain from three independent cultures.

### Analysis of sterol composition.

Cultures were grown in YPD (1% yeast extract, 2% peptone, 2% glucose) medium to facilitate comparisons with data from a previously published sterol analysis of the S. cerevisiae Δ*spf1* mutant using this medium (25). A total of 1 × 10^7^ conidia were inoculated into 5 ml of YPD medium in a 50-ml conical tube and incubated at 37°C for 24 h with gentle shaking (200 rpm). The biomass was washed with sterile distilled water to remove sterol impurities and dried under a vacuum. The dried mycelium was saponified in 1 ml of alcoholic KOH (3% KOH in ethanol). Sterols were extracted into petroleum ether (hexane). The sterol concentrations were analyzed by gas chromatography using purchased standards for ergosterol and lanosterol to identify peaks of interest. Values are presented as percentages of total sterols.

### Animal models of invasive aspergillosis.

On day −1, groups of 12 male (26 to 34 g) or female (23 to 30 g) CF-1 outbred mice (Charles River) were immunosuppressed with a single dose of 40 mg/kg of body weight of triamcinolone acetonide (TA) injected subcutaneously. On day 0, mice were anesthetized using 3.5% isoflurane and intranasally inoculated with 2 × 10^6^ conidia from the selected strain contained in 20 μl of saline. A saline control group without fungus was monitored in parallel. Survival was tracked for the next 14 days. For histopathological analysis of murine lung tissues, female CF-1 mice treated as described above were sacrificed on day 3 postinfection. Lungs were fixed in a 10% neutral buffered formalin solution (Sigma) for 48 h. The samples were dehydrated, embedded in paraffin, sectioned at 5 μm, and stained with Gomori’s methenamine silver (GMS) or hematoxylin and eosin (HE). Pictures were taken using a 7.4 Slider RTKE Spot camera attached to an Olympus BX51 microscope. For the insect model, groups of at least 25 similarly sized larvae of G. mellonella were infected in the right last proleg with 20 μl of phosphate-buffered saline (PBS) containing 2 × 10^5^ or 1 × 10^6^ conidia using U-100 insulin syringes (28G1/2; Becton, Dickinson). Larvae were kept for 7 days at 37°C in the dark and monitored daily. Larvae were scored as dead upon dark-brown pigmentation and loss of motility.

### Ethics statement.

Mouse studies were performed in agreement with the recommendations in the *Guide for the Care and Use of Laboratory Animals* of the National Research Council (63). Our animal use protocol was approved by the Institutional Animal Care and Use Committee (IACUC) at the University of Cincinnati.

### Statistical analysis.

Statistical data analysis was performed with GraphPad Prism. Unpaired, two-tailed Student’s *t* tests or one-way analyses of variance (ANOVAs) with Dunnett’s or Tukey’s multiple-comparison tests were used for growth-related phenotypes, gene expression, and cell wall data. Differences in mortality curves were assessed using log rank (Mantel-Cox) tests.

The approach taken by CLC Genomics Workbench to calculate gene expression levels is based on methods described previously (64). We used the Bonferroni adjustment (*P < *0.05) as the cutoff point for the selection of differentially expressed genes, resulting in fold changes above 1.38.

### Data availability.

The data sets generated were deposited in the Gene Expression Omnibus (GEO) (65) with the accession number GSE179173 (https://www.ncbi.nlm.nih.gov/geo/query/acc.cgi?acc=GSE179173).
