# Supplementary material for: Pleiotropic Effects of the P5-Type ATPase SpfA on Stress Response Networks Contribute to Virulence in the Pathogenic Mold Aspergillus fumigatus
Source: mBio. 2021 Oct 19;12(5):e02735-21. doi: 10.1128/mBio.02735-21 (PMC8524344; doi:10.1128/mBio.02735-21)
Supplement: FIG S1 [file mbio.02735-21-sf001.pdf]

```
                   NTD
SpfA-Af        1  MLR---AKLVDDPQIKYASL--------------------------------------HN
Spf1-Sc        1  MTK---KSFVSSPIVRDSTL--------------------------------------LV
Spf1-Ca        1  MS-----DLVANPAIQGAEL--------------------------------------LV
Spf1-Po        1  MAPPPSPALVRSSQIKSAEL--------------------------------------LR
Spf1-Bb        1  MAP-----LVENAQIKHAQL--------------------------------------LN
ATP13A1-Hs     1  MAA---AAAVGNAVPCGARPCGVRPDGQPKPGPQPRALLAAGPALIANGDELVAAVWPYR

                               TMa                                 TMb
SpfA-Af       20  PLPTQLHAYVWPFL-IIWPAFFAVYLSP--ERYDTYIQGQEWTFVWSGSIITAQSLLWLM
Spf1-Sc       20  PKSLIAKPYVLPFF-PLYATFAQLYF----QQYDRYIKGPEWTFVYLGTLVSLNILVMLM
Spf1-Ca       18  PKAVFLRPYVWPFS-IIYPIFLQIYF----QQYDKYIGGKEWTFVYTIAIVSVNLLFWLM
Spf1-Po       23  PLAWYSHAYVWPFA-IAWPVFLRFYLSA--DLYEKHIGGPEWTFVWLGTIITFQSLAWLT
Spf1-Bb       18  PLSFHFRAYIWPFA-IIWPIFLRYFLTP--ELYEKHIGAPEWTFVWVGSIITVQSLVWLS
ATP13A1-Hs    58  RLALLRRLTVLPFAGLLYPAWLGAAAAGCWGWGSSWVQIPEAALLVLATICLAHALTVLS

SpfA-Af       77  TKWNINIQTLFTATKARSLDSAQLIKVIPVANAGSAEICPLHCDTM-GGKKTFSFLFQKR
Spf1-Sc       75  PAWNVKIKAKFNYSTTKNVNEATHILIYTTPNNGSDGIVEIQRVTE-AGSLQTFFQFQKK
Spf1-Ca       73  PHWNIDINAKFNYTKVDKISDASYIKITPAPNSGMGEICEINRETFHDGEKQVSFLYQKR
Spf1-Po       80  TKWSVNLRAIFTARTVKGVEDAELIKLIPIANAGSADICKIERDQV-GGKNNISFLFQKR
Spf1-Bb       75  THWSVNLDAILTAQSAKSVEDAQLIKVIPVANAGSAEICKLVRDKT-GGKTNLSFLFQKR
ATP13A1-Hs   118  GHWSVHAHCALTCTPEYDPSKATFVKVVPTPNNGSTELVALHRNEGEDGLEVLSFEFQKI

                        NTD            A domain             A domain
SpfA-Af      136  RFLYYPE-RQCFAPLSYVLDAEPKPPVKVFQQAQGLTSKEEIDRIQHHYGDNTFDIPVPT
Spf1-Sc      134  RFLWHEN-EQVFSSPKFLVDESP--KIGDFQKCKGHSG--DLTHLKRLYGENSFDIPIPT
Spf1-Ca      133  RYLFHSK-IGKFSPPEFVFDEAP--KLAVYQNTKGLSG--DLEKMIRNYGSNRFDIPIPT
Spf1-Po      139  RFLYDTT-DKSFKTLEYDIDAEPKPKIEKFQLSKGITSATELTRIEQHYGTNTFDIPVPS
Spf1-Bb      134  RFLYNPD-TKSFDTLKYAIDSEPQPLIRDFQFSRGIEQKSELSRMEHHYGTNTFDIPVPT
ATP13A1-Hs   178  KYSYDALEKKQFLPVAFPVGN----AFSYYQSNRGFQEDSEIRAAEKKFGSNKAEMVVPD

                                 TM1                      TM2
SpfA-Af      195  FMELFKEHAVAPFFVFQVFCVGLWMLDEYWYYSLFTLFMLVVFESTVVWQRQRTLNEFRG
Spf1-Sc      189  FMELFKEHAVAPLFVFQVFCVALWLLDEFWYYSLFNLFMIISMEAAAVFQRLTALKEFRT
Spf1-Ca      188  FMELFKEHAVAPFFVFQIFCVALWCMDEQWYYSLFSLFMLVSFEMTTVFQRRTTMAEFQS
Spf1-Po      198  FTELFQEHAVAPFFVFQIFCVGLWMLDDYWYYSLFTLVMLVAFESTVVWQRQRTLNEFRG
Spf1-Bb      193  FTELFKEHAVAPFFVFQIFCVGLWMLDDYWYYSLFTLFMLVAFESTVVWQRQRTLTEFRG
ATP13A1-Hs   234  FSELFKERATAPFFVFQVFCVGLWCLDEYWYYSVFTLSMLVAFEASLVQQQMRNMSEIRK

                     A domain
SpfA-Af      255  MNIKPYDVWVYRQKKWQELTSDKLLPGDLMSVNRTKEDSGVACDILLIEGSAIVNEAMLS
Spf1-Sc      249  MGIKPYTINVFRNKKWVALQTNELLPMDLVSITRTAEESAIPCDLILLDGSAIVNEAMLS
Spf1-Ca      248  MGIKPYDVYAYRDGKWVKIPTTDLLPGDLISITRTNEGSALPCDLLLVDGSAIVNEAMLS
Spf1-Po      258  MSLKPYDMWVYRLGKWVEVQSDKLVPGDLVSVNRSKEDGGVACDMLLVEGTAIVNEAMLS
Spf1-Bb      253  MSIKPYDMWVYRLGAWTEVQSDALLPGDLVSVTRTKDDSGVACDMILVEGTAIVNEAMLS
ATP13A1-Hs   294  MGNKPHMIQVYRSRKWRPIASDEIVPGDIVSIGRSPQENLVPCDVLLLRGRCIVDEAMLT

SpfA-Af      315  GESTPLLKESIQLRPGDDLIDPDGLDKNAFVHGGTKVLQITHHNSNGEDGSEKARKLSSG
Spf1-Sc      309  GESTPLLKESIKLRPSEDNLQLDGVDKIAVLHGGTKALQVTP------------PEHKSD
Spf1-Ca      308  GESTPLLKESIKLRPADEQLQPEGFDKNSILHGGTMALQVTK------------PES-PI
Spf1-Po      318  GESTPLLKESIQLRPGDAQLEPEGLDKNSFLWGGTKVLQITHGTADEA-----RPKTASG
Spf1-Bb      313  GESTPLLKDSIQLRPADATLDAEGLDKNAFLWGGTKVLQITHASADQE-----KPVLPSG
ATP13A1-Hs   354  GESVPQMKEPIEDLSPDRVLDLQADSRLHVIFGGTKVVQHIP------------PQKATT
                 (S/T)GES
                                              A domain                 TM3
SpfA-Af      375  VPLPPDNGAVGVVVKTGFETSQGSLVRTMIYSTERVSANNVEALLFILFLLIFAIAAAWY
Spf1-Sc      357  IPPPPDGGALAIVTKTGFETSQGSLVRVMIYSAERVSVDNKEALMFILFLLIFAVIASWY
Spf1-Ca      355  VPVAPDNGAFAVVTKTGFETSQGSLVRMMIFSSERVSVGNKEAFFFILFLLIFAIAASWY
Spf1-Po      373  VPRPPDDGAMAIVTKTGFETSQGSLVRTMIYSTERVSANNVEALLFILFLLIFALAASWY
Spf1-Bb      368  VPPPPDNGAMAIVTKTGFETSQGSLVRTMIYSTEHVSANNVEALLFILFLLIFAIAASWY
ATP13A1-Hs   402  GLKPVDSGCVAYVLRTGFNTSQGKLLRTILFGVKRVTANNLETFIFILFLLVFAIAAAAY
```

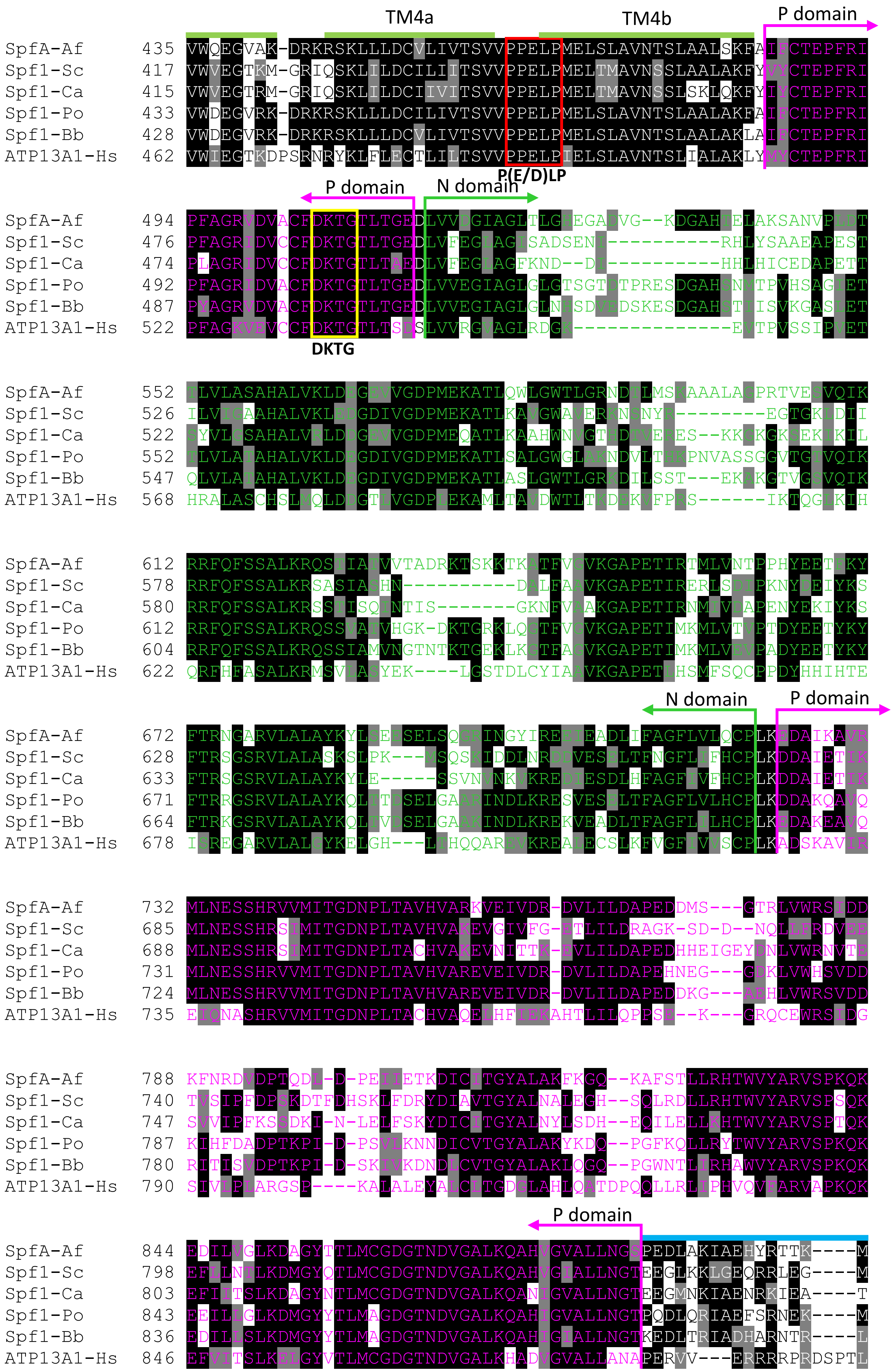
TM4a
TM4b
P domain
P(E/D)LP
P domain
N domain
DKTG
N domain
P domain
P domain
SpfA-Af
Spf1-Sc
Spf1-Ca
Spf1-Po
Spf1-Bb
ATP13A1-Hs

```
                                              Arm
SpfA-Af      900 KEIYEKQVSMMQRFNQPPPPVPVQIAHLYPPGPRNPHYQKAMEREAQRKGAATLATAGNQ
Spf1-Sc      854 KMMYIKQTEFMARWNQPQPPVPEPIAHLFPPGPKNPHYLKALESKG----TVITPEIRKA
Spf1-Ca      859 LKVYEKQSQIFNNWGKPAPPVPAIIAHLYPPGPLNPKYLEAMEKKG----VTITDDMRKA
Spf1-Po      899 KQMYQKQIDLMVRFNQPTPPVPVMIAHLYPPGPSNPHYMKAMEREAKNKGVTVEELIKAS
Spf1-Bb      892 KDMYQKQCDLMKRFNQPQPPAPVLIAHLYPPGPANPHYQKAVEREAEKKGVSVQEYVKLA
ATP13A1-Hs   903 SNSGIRATSRTAKQRSGLP-----------------------------------------

                                              Arm
SpfA-Af      960 -----TEHIPTITSPGAQALQQSNANLTPQQQRQQQASIAAAGFADKLTSSMLEQELDDS
Spf1-Sc      910 VEEANSKPVEVIK-------------------PNGLSEKKPADLASLLLNSAGDAQ--GD
Spf1-Ca      915 VVEAMKEPVKVPE-----------------KNAANGGFNTNSNFADTILGAMNDAEA-ED
Spf1-Po      959 -----GHPTETITSAGARELLKSD----PNQ-NLGPAAKKAAGLADKLTAQMMESELDDG
Spf1-Bb      952 -----GHSLETITTPAAQQLINSN---DPQAARQAAAQAKAASFADKLSSGMMEAELGDD
ATP13A1-Hs   922 -----------------------------------PSEEQPTSQRDRLSQVLRDLE--DE

                        P domain                   TM5
SpfA-Af     1015 EPPTIKLGDASVAAPFTSKLANVIAIPNILRQGRCTLVATIQMYKILALNCLISAYSLSV
Spf1-Sc      949 EAPALKLGDASCAAPFTSKLANVSAVTNIIRQGRCALVNTIQMYKILALNCLISAYSLSI
Spf1-Ca      957 EAPVLKLGDASVAAPFTSKLANVNTVTHIIRQGRVALVSTIQMYKILALNCLISSYSLSV
Spf1-Po     1009 EPPSLKLGDASVAAPFTSKLRNVIAVPNIIRQGRCTLVATIQMYKILALNCLITAYSLSV
Spf1-Bb     1004 EPPTLKLGDASVAAPFTSKLRDVIAIPNIIRQGRCTLVATIQMYKILALNCLISAYSLSV
ATP13A1-Hs   945 STPIVKLGDASIAAPFTSKLSSIQCICHVIKQGRCTLVTTLQMFKILALNALILAYSQSV

                           TM6
SpfA-Af     1075 IYLDGIKFGDGQVTISGMLMSVCFLSISRAKSVEGLSKERPQPNIFNVYIIGSVLGQFAI
Spf1-Sc     1009 IYMAGVKFGDGQATVSGLLLSVCFLSISRGKPLEKLSKQRPQSGIFNVYIMGSILSQFAV
Spf1-Ca     1017 LYLAGMKFGDGQATISGILLSVCFLSISRGRPLEKLSKERPQDGIFNIYIMGSILGQFAV
Spf1-Po     1069 LYLEGIKFGDGQYTISGMLMSVCFLSISRARSVEGLSKERPQPNIFNFYIIGSILGQFAV
Spf1-Bb     1064 LYLEGIKFGDTQYTISGMLMSVCFLSISRAKVVEGLSKERPQPNIFNIYIIGSILGQFAV
ATP13A1-Hs  1005 LYLEGVKFSDFQATLQGLLLAGCFLFISRSKPLKTLSRERPLPNIFNLYTILTVMLQFFV

                   TM7                                 TM8
SpfA-Af     1135 HIATLIYLSNYVYSIEPRK--SDIDLEGEFEPSLLNSAIYLLQLIQQISTFSINYQGRPF
Spf1-Sc     1069 HIATLVYITTEIYKLEPRE--PQVDLEKEFAPSLLNTGIFIIQLVQQVSTFAVNYQGEPF
Spf1-Ca     1077 HIITLIYITREIYILEPRE--PKVDLEKEFSPSLLNTGMFLLQLAQQVSTFAVNYIGLPF
Spf1-Po     1129 HIFTLIYIARFCDKIAPRT--ESVDLEAEFSPSLLNSAVYLLQLIQQISTFAINYQGRPF
Spf1-Bb     1124 HIVTLIYVARLSEKIEPRS--DDVDLEAEFEPSLLNSAIYLLQLVQQVSTFAINYQGRPF
ATP13A1-Hs  1065 HFLSLVYLYREAQARSPEKQEQFVDLYKEFEPSLVNSTVYIMAMAMQMATFAINYKGPPF

                          TM9                                   TM10
SpfA-Af     1193 RESIRENKAMYWGLVAASGVAFS-CATEFIPELNEKMRLVPFSTEFKVTLTVLMIIDYAG
Spf1-Sc     1127 RENIRSNKGMYYGLLGVTGLALA-SATEFLPELNEAMKFVPMTDDFKIKLTLTLLLDFFG
Spf1-Ca     1135 RESITSNKGMYYGLLGVAGLTFS-CSTEFIPELNEVMQFVPMTIDFKTKLTGCIILDLVV
Spf1-Po     1187 RESISENRGMYWGIIGVSGIAFA-CSTEILPELNEQMKLVPFTAEFRQTLTGIMVLDYGA
Spf1-Bb     1182 REALTENKAMFYGILGVSGLAFV-CAMELIPEINESIKLVPFTEEFKLKMTVVMALDFIV
ATP13A1-Hs  1125 MESLPENKPLVWSL-AVSLLAIIGLLLGSSPDFNSQFGLVDIPVEFKLVIAQVLLLDFCL

SpfA-Af     1252 CWIIENVLKNLFSDFRPKDIAVRRPDQLQREMERKKQEELETQAEKERQRKV--------
Spf1-Sc     1186 SWGVEHFFKFFFMDDKPSDISVQQVKIASK------------------------------
Spf1-Ca     1194 TFAIEYVLKYFFMNSKAADIALREEDEVDA------------------------------
Spf1-Po     1246 CWIIEVVFKYLFSDLKARDIAERRPDQLERERVRKEAEL-KIKMEEDDKKRIQEVEEYER
Spf1-Bb     1241 CWVIEVVLKSLFSDYRPRDIAERRPDQLAREAARKKIVA-DEKAIEEEKKRLEKVAEFER
ATP13A1-Hs  1184 ALLADRVLQFFLGTPKLKVPS---------------------------------------

SpfA-Af          ----------------------------
Spf1-Sc          ----------------------------
Spf1-Ca          ----------------------------
Spf1-Po     1305 KMEAKKRELAE-RWGVQLPERGQPAAAR
Spf1-Bb     1300 KVEERKRQLQEWRAGRQRQ---------
ATP13A1-Hs       ----------------------------
```
