# Supplementary figures and images for: Pleiotropic Effects of the P5-Type ATPase SpfA on Stress Response Networks Contribute to Virulence in the Pathogenic Mold Aspergillus fumigatus
Source: mBio. 2021 Oct 19;12(5):e02735-21. doi: 10.1128/mBio.02735-21 (PMC8524344; doi:10.1128/mBio.02735-21)

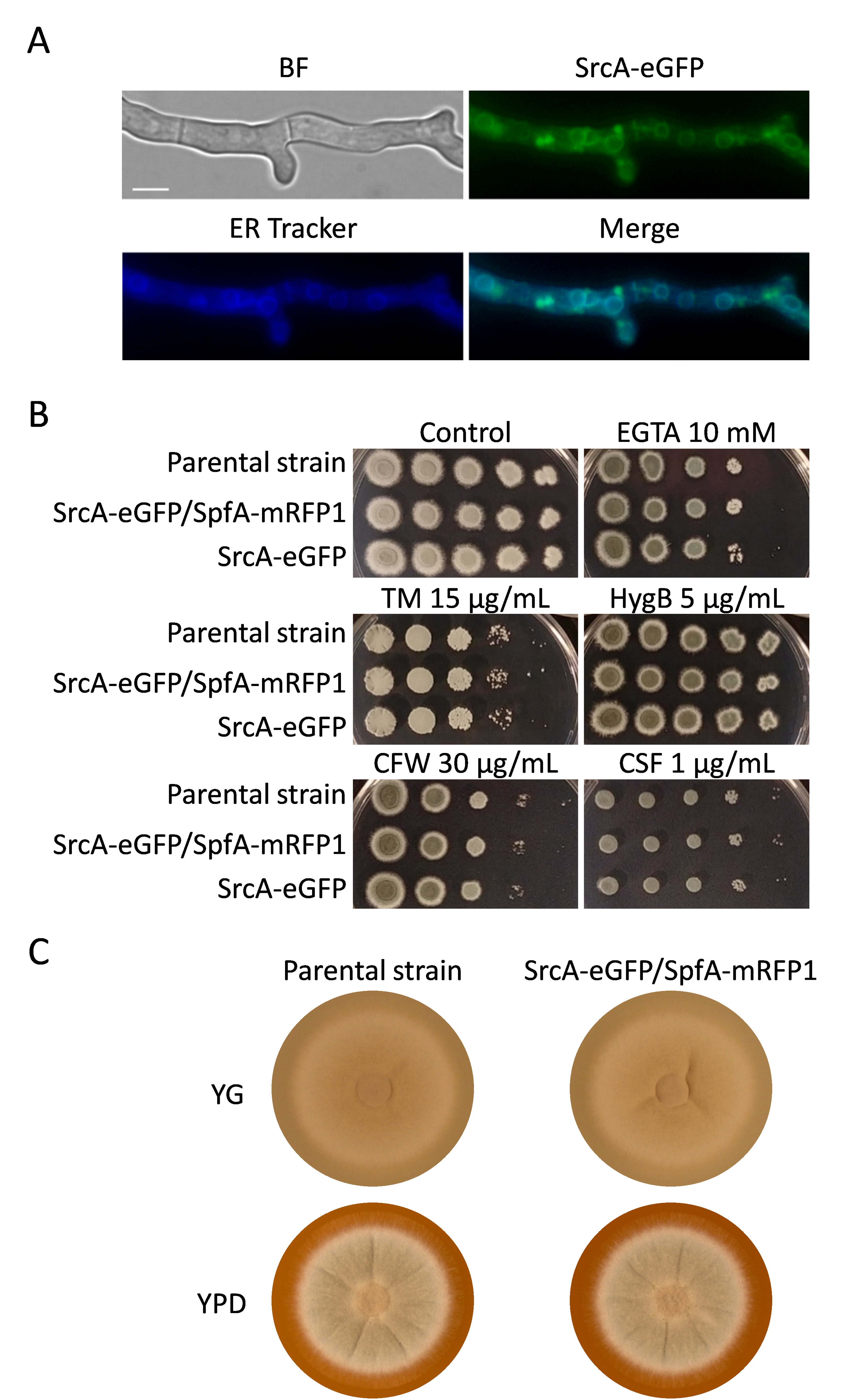

Supplement: FIG S2 [file mbio.02735-21-sf002.jpg]

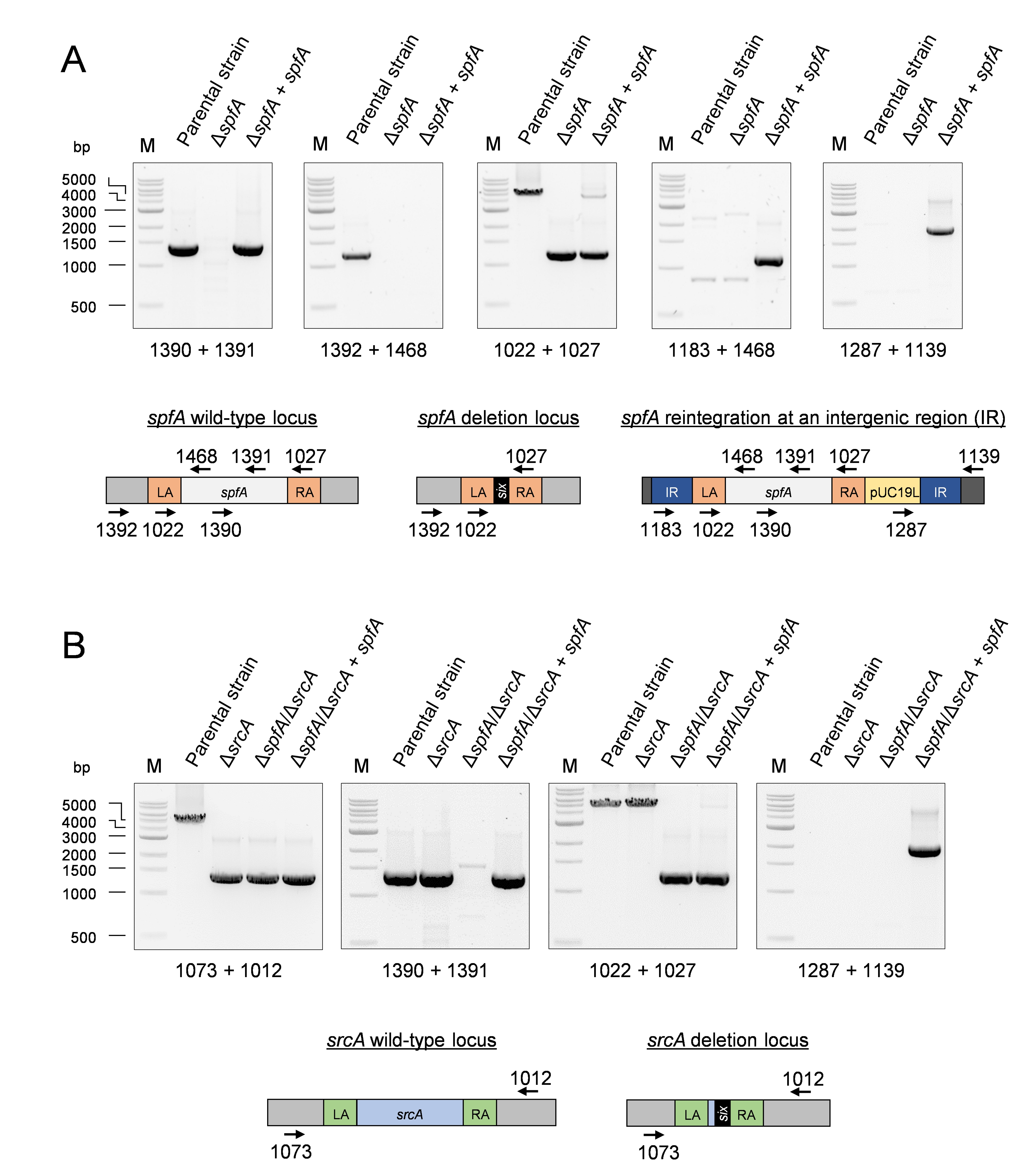

Supplement: FIG S3 [file mbio.02735-21-sf003.jpg]

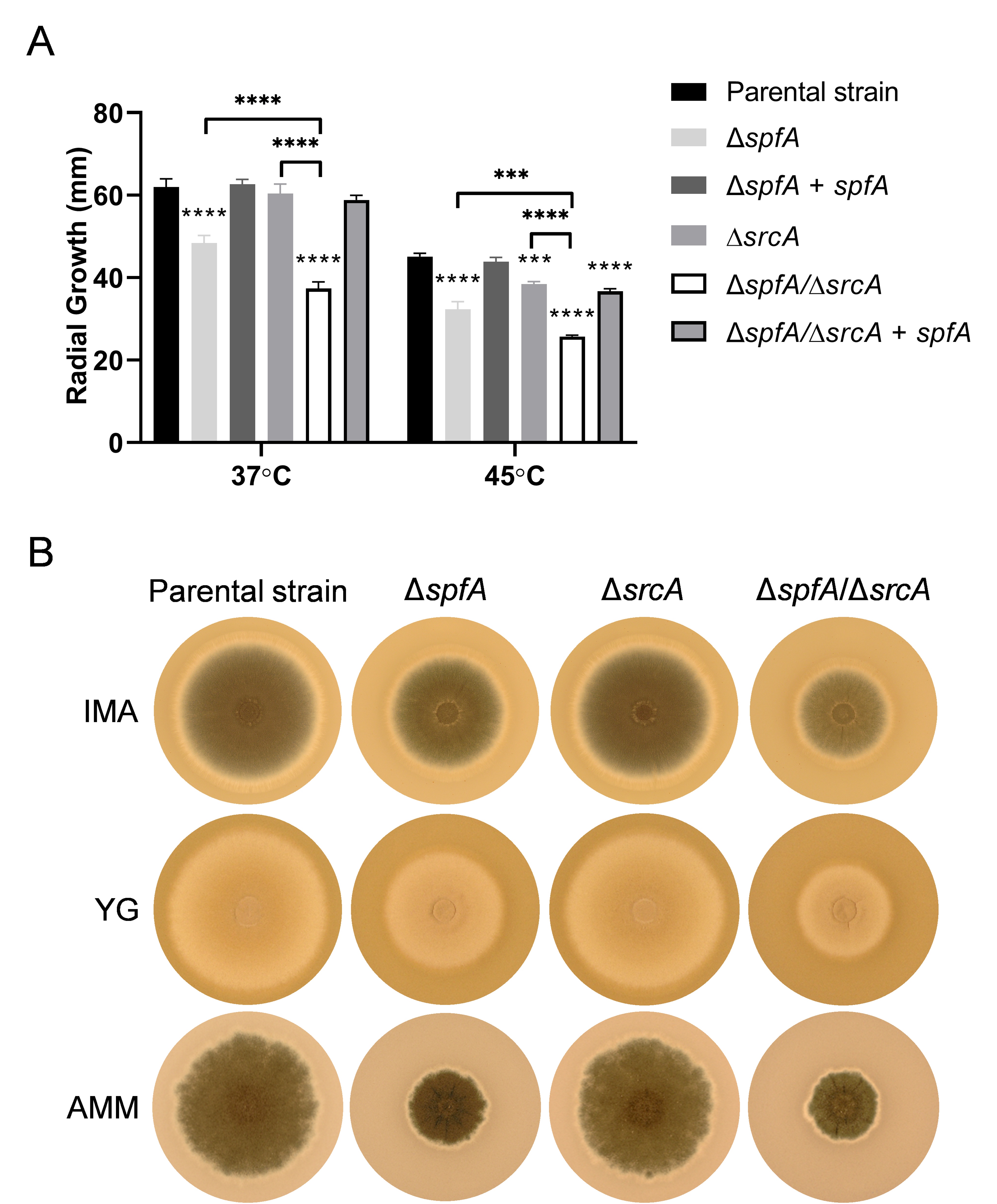

Supplement: FIG S4 [file mbio.02735-21-sf004.jpg]

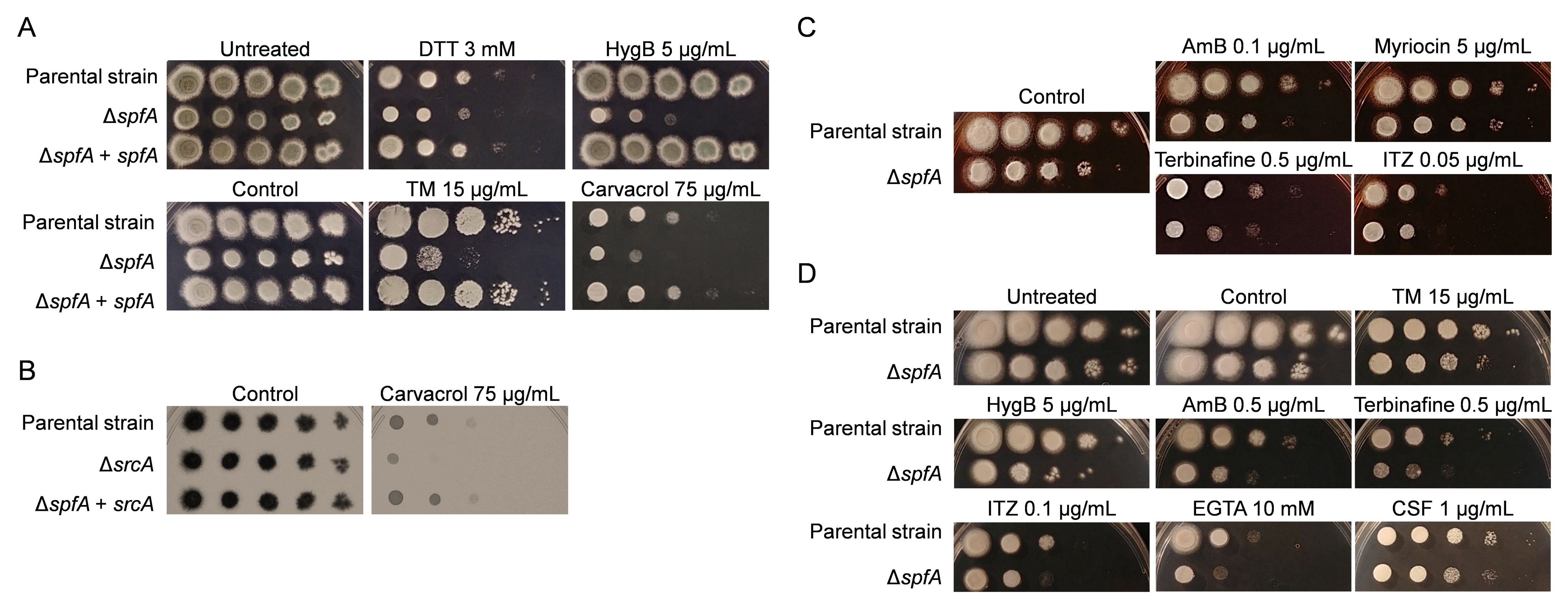

Supplement: FIG S5 [file mbio.02735-21-sf005.jpg]

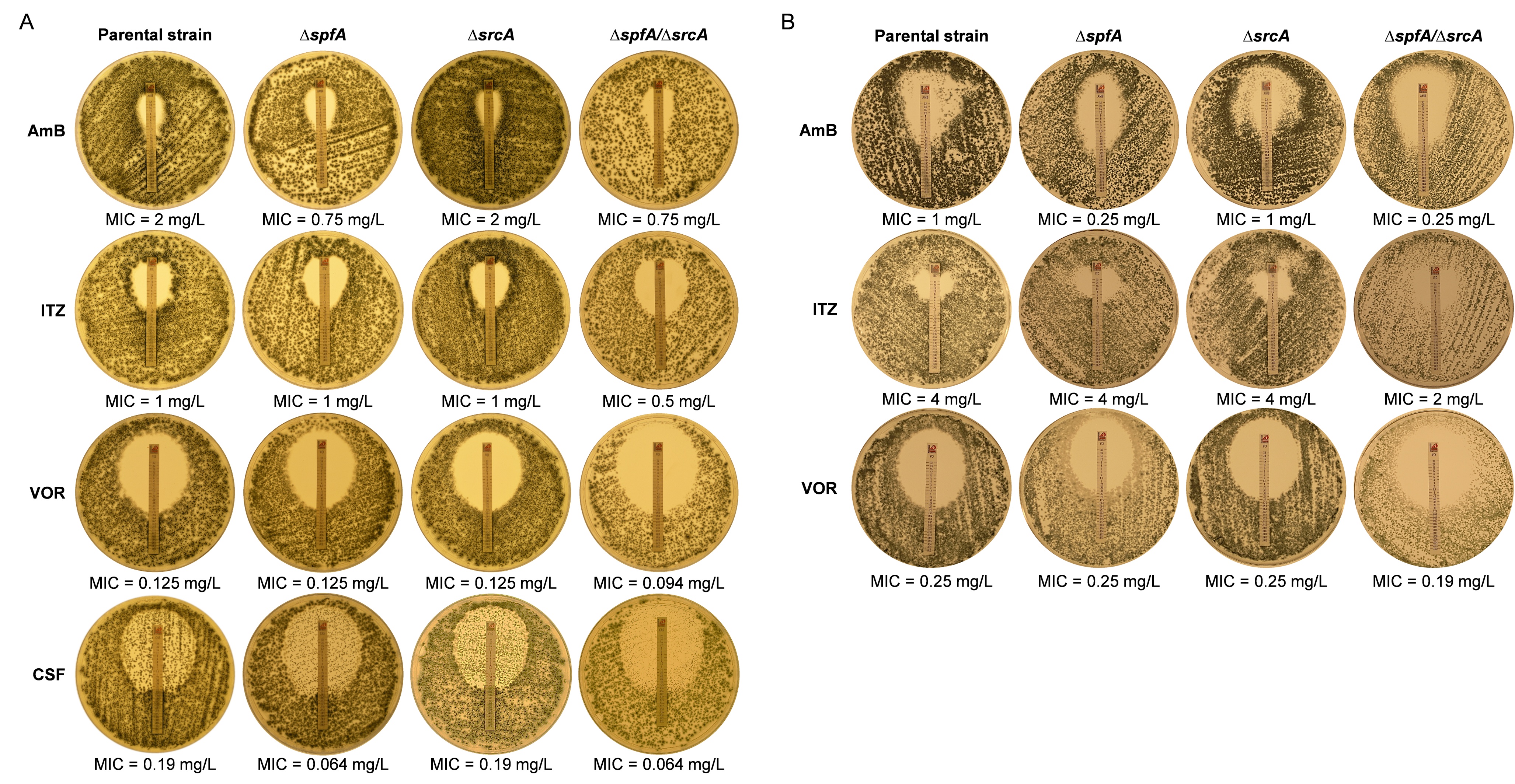

Supplement: FIG S6 [file mbio.02735-21-sf006.jpg]

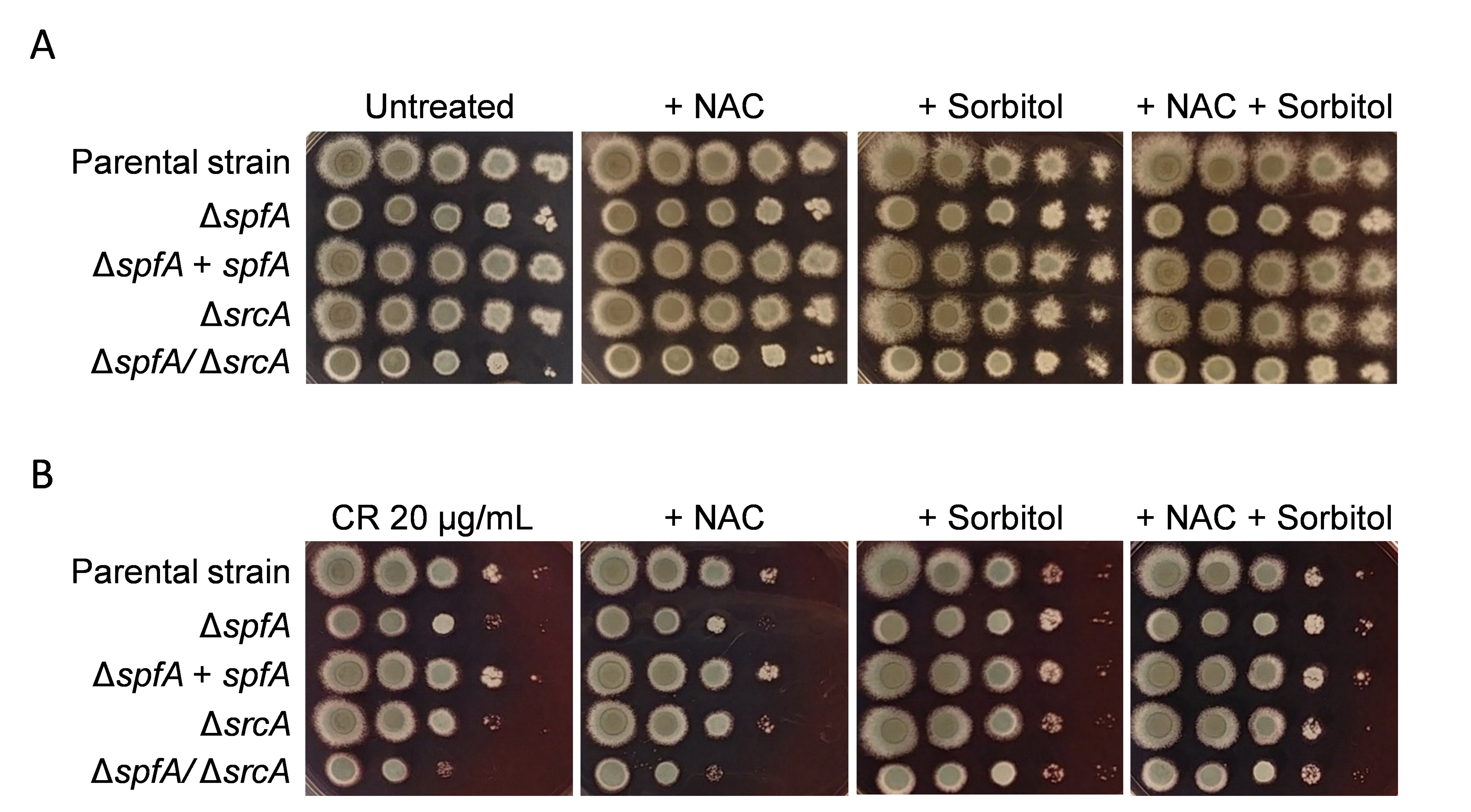

Supplement: FIG S7 [file mbio.02735-21-sf007.jpg]

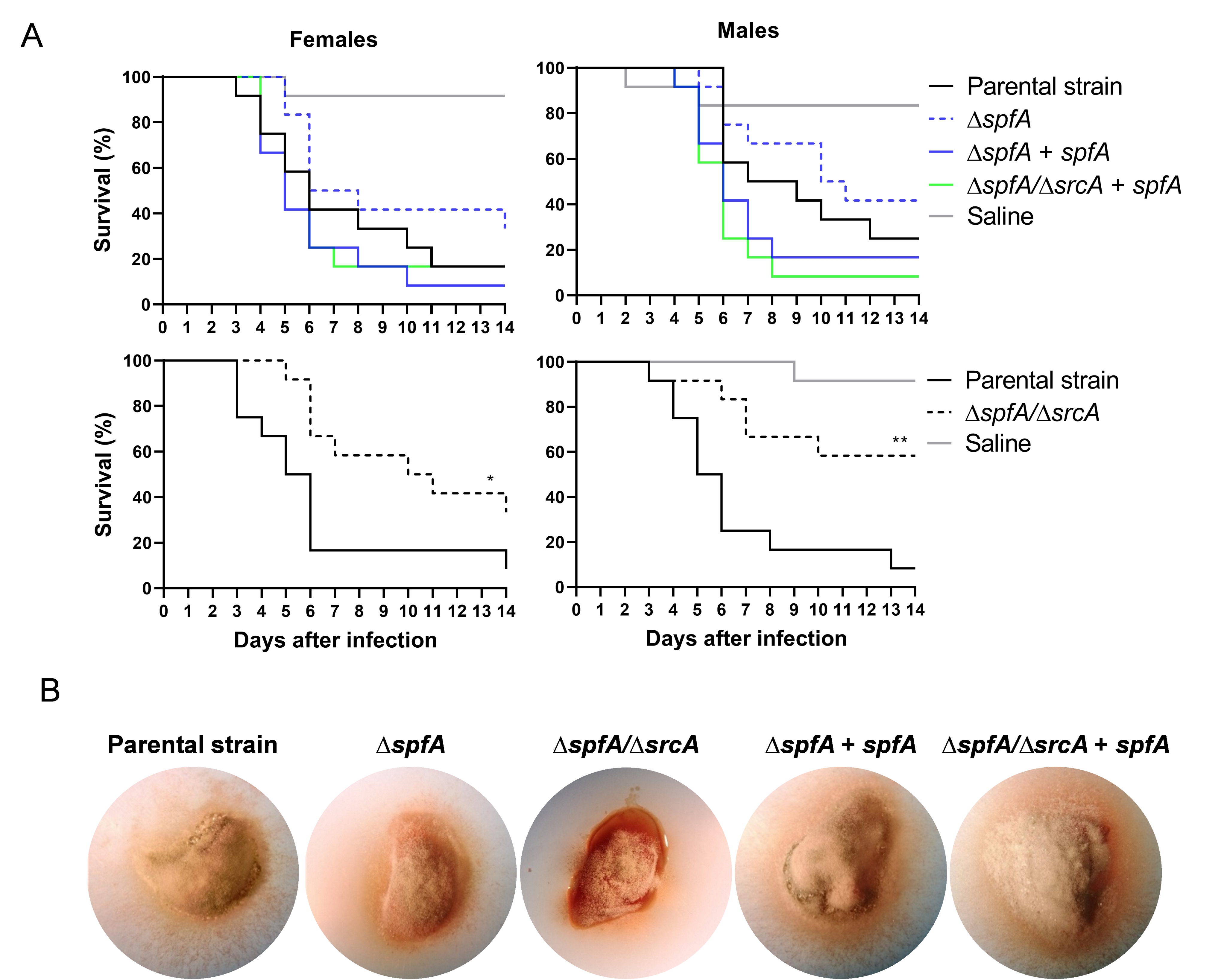

Supplement: FIG S8 [file mbio.02735-21-sf008.jpg]
