# Supplementary material for: Pleiotropic Effects of the P5-Type ATPase SpfA on Stress Response Networks Contribute to Virulence in the Pathogenic Mold Aspergillus fumigatus
Source: mBio. 2021 Oct 19;12(5):e02735-21. doi: 10.1128/mBio.02735-21 (PMC8524344; doi:10.1128/mBio.02735-21)
Supplement: TABLE S1 [file mbio.02735-21-st001.docx]

**TABLE S1** Strains of *Aspergillus fumigatus* used in this study.

| **Strain** | **Number** | **Genotype** | **Origin** |
| --- | --- | --- | --- |
| KU70 | 124 | ∆*akuA*::*ptrA* | * |
| Δ*hacA* | 144 | ∆*akuA*::*ptrA*, ∆*hacA*::*hph* | Reference 9 |
| KU80 | 399 | ∆*akuB*::*pyrG^+^* | Reference 59 |
| SrcA-eGFP | 725 | ∆*akuB*::*pyrG^+^*_,_ *srcA*::*egfp-six* | Reference 19 |
| SrcA-eGFP/ SpfA-mRFP1 | 826 | ∆*akuB*::*pyrG^+^*_,_ *srcA*::*egfp-six, spfA*::*mrfp1-six* | This study |
| Δ*spfA* | 772 | ∆*akuB*::*pyrG^+^*_,_ ∆*spfA*::*six* | This study |
| Δ*spfA* + *spfA* | 818 | ∆*akuB*::*pyrG^+^*_,_ ∆*spfA*::*six, spfA* | This study |
| Δ*srcA* | 402 | ∆*akuB*::*pyrG^+^*_,_ ∆*srcA*::*six* | Reference 19 |
| Δ*srcA + srcA* | 568 | ∆*akuB*::*pyrG^+^*_,_ ∆*srcA*::*srcA* | Reference 19 |
| Δ*srcA/*Δ*spfA* | 756 | ∆*akuB*::*pyrG^+^*_,_ ∆*srcA*::*six,* ∆*spfA*::*six* | This study |
| Δ*srcA/*Δ*spfA* + *spfA* | 816 | ∆*akuB*::*pyrG^+^*_,_ ∆*srcA*::*six,* ∆*spfA*::*six, spfA* | This study |

*Krappmann S, Sasse C, Braus GH. 2006. Gene targeting in Aspergillus fumigatus by homologous recombination is facilitated in a nonhomologous end-joining-deficient genetic background. Eukaryot Cell 5:212–215.
