# Supplementary material for: Pleiotropic Effects of the P5-Type ATPase SpfA on Stress Response Networks Contribute to Virulence in the Pathogenic Mold Aspergillus fumigatus
Source: mBio. 2021 Oct 19;12(5):e02735-21. doi: 10.1128/mBio.02735-21 (PMC8524344; doi:10.1128/mBio.02735-21)
Supplement: TABLE S2 [file mbio.02735-21-st002.docx]

**TABLE S2** List of oligonucleotides used in this study.

| **Number** | **Gene/locus/purpose** | **Nucleotide sequence (5’-3’)** |
| --- | --- | --- |
| **Molecular cloning** | | |
| 1053 | Marker module (*cme^R^* with β-*rec*) | GCATCACTCAGGTCCTATAGGTC |
| 1054 | Marker module (*cme^R^* with β-*rec*) | GCACTAGATGGACCATATTATGC |
| 1061 | pUC19L | GGCATGCAAGCTTGGCGTAATCATGG |
| 1062 | pUC19L | GTACCGAGCTCGAATTCACTGGCCGT |
| 1434 | *spfA-mrfp1* | AATTCGAGCTCGGTACTGTTCCTGTCCAGATTGCG |
| 1435 | *spfA-mrfp1* | CGTCCTCGGAGGAGGCCATGACCTTCCTCTGTCGTTCC |
| 1436 | *spfA-mrfp1* | TGGTCCATCTAGTGCGATGTTGGTTACCACAGGG |
| 1437 | *spfA-mrfp1* | GCCAAGCTTGCATGCCGATTCACTCTTTCACGGTGATGCC |
| 1438 | *mrfp1* | ATGGCCTCCTCCGAGGACG |
| 1439 | *mrfp1* | AGGACCTGAGTGATGCttaGGCGCCGGTGGAGTGGCG |
| 1386 | ∆*spfA* | AATTCGAGCTCGGTACAACAACGGACATCAGTATCTGG |
| 1387 | ∆*spfA* | AGGACCTGAGTGATGCTGATGATGTGGGACTAACATGG |
| 1388 | ∆*spfA* | ATGGTCCATCTAGTGCCTTTTTGCGTTAGAATGATGTGGG |
| 1389 | ∆*spfA* | GCCAAGCTTGCATGCCTCGGTACGATAGTCCATGTGCTGG |
| 1432 | ∆s*pfA* complementation | aatgtattaattaaAACAACGGACATCAGTATCTGG |
| 1433 | ∆s*pfA* complementation | aatgtaggcgcgccTCGGTACGATAGTCCATGTGCTGG |
| 1309 | pUC19L+ Intergenic region | aatgtaggcgcgccGGCATGCAAGCTTGGCGTAATCATGG |
| 1310 | pUC19L+ Intergenic region | aatgtattaattaaTGCACGCTACATATGCACGCATCG |
| **Confirmatory PCR analysis** | | |
| 1022 | *spfA* | GTCGAGGCATCTTTGGGTATAA |
| 1027 | *spfA* | ATCCCTTCGTTACGTCCAATC |
| 1390 | *spfA* | CTAATGTCCCACTCGACACGACCC |
| 1391 | *spfA* | ACCAGGGTACAACGACCTTGACGC |
| 1392 | *spfA* | TCATCGTTCGCATCCCTTACCTCC |
| 1468 | *spfA* | AGGCATGAAGTTGAGTAGGG |
| 1012 | *srcA* | GCACAGGCCATCGTCTATATC |
| 1073 | *srcA* | GGCATCATCCTACTGTCTAAATCC |
| 1139 | Intergenic Region | CAACCTGCATGGCTCTTAATC |
| 1183 | Intergenic Region | GCAGAGATTCGGCACCGGCTAGTGG |
| 1287 | pUC19L | CGCCAGGGTTTTCCCAGTCACGAC |
| **Gene expression analysis (RT-qPCR)** | | |
| 1252 | *18S rRNA* (F) | ACTGATACGGGGCTCTTTTG |
| 1253 | *18S rRNA* (R) | GACTTGCCCTCCAATTGTTC |
| 1378 | *spfA* (F) | GGGTGTATCGCCAAAAGAAG |
| 1379 | *spfA* (R) | TCACAAGCAACGCCACTATC |
| 1232 | *srcA* (F) | CGGTGCTGCTACTGTTTTTG |
| 1233 | *srcA* (R) | ACTTGTGGAAATGGGACAGC |
| 1218 | *bipA* (F) | TGATGAAGAGCGTCTGGTTG |
| 1219 | *bipA* (R) | TCTGGACATCCTTGTCATCG |
| 1271 | *pdiA* (F) | TCAAGGTCGATTGCACTGAG |
| 1272 | *pdiA* (R) | GGAGGCAAAGTAACCGATGA |
| 1279 | *eroA* (F) | GAGTTCCGCAATCGCTTTAG |
| 1280 | *eroA* (R) | TTCAGAGCCGTTCCATATCC |
| 1355 | *hacA^u/i^* (F) | TTGTTCAAGCAAGAAGGTGATG |
| 1356 | *hacA^u^* (R) | GTCGCACAACACCGCTG |
| 1354 | *hacA^i^* (R) | ACTGACACTGCAGGATGTTGTG |
